# Persistent Gastric Colonization with *Burkholderia pseudomallei* and Dissemination from the Gastrointestinal Tract following Mucosal Inoculation of Mice

**DOI:** 10.1371/journal.pone.0037324

**Published:** 2012-05-18

**Authors:** Andrew Goodyear, Helle Bielefeldt-Ohmann, Herbert Schweizer, Steven Dow

**Affiliations:** 1 Department of Microbiology, Immunology, and Pathology, Rocky Mountain Regional Center for Excellence for Biodefense and Emerging Infectious Diseases Research, Colorado State University, Fort Collins, Colorado, United States of America; 2 School of Veterinary Science, University of Queensland, Queensland, Australia; Tulane University School of Medicine, United States of America

## Abstract

Melioidosis is a disease of humans caused by opportunistic infection with the soil and water bacterium *Burkholderia pseudomallei*. Melioidosis can manifest as an acute, overwhelming infection or as a chronic, recurrent infection. At present, it is not clear where *B. pseudomallei* resides in the mammalian host during the chronic, recurrent phase of infection. To address this question, we developed a mouse low-dose mucosal challenge model of chronic *B. pseudomallei* infection and investigated sites of bacterial persistence over 60 days. Sensitive culture techniques and selective media were used to quantitate bacterial burden in major organs, including the gastrointestinal (GI) tract. We found that the GI tract was the primary site of bacterial persistence during the chronic infection phase, and was the only site from which the organism could be consistently cultured during a 60-day infection period. The organism could be repeatedly recovered from all levels of the GI tract, and chronic infection was accompanied by sustained low-level fecal shedding. The stomach was identified as the primary site of GI colonization as determined by fluorescent *in situ* hybridization. Organisms in the stomach were associated with the gastric mucosal surface, and the propensity to colonize the gastric mucosa was observed with 4 different *B. pseudomallei* isolates. In contrast, *B. pseudomallei* organisms were present at low numbers within luminal contents in the small and large intestine and cecum relative to the stomach. Notably, inflammatory lesions were not detected in any GI tissue examined in chronically-infected mice. Only low-dose oral or intranasal inoculation led to GI colonization and development of chronic infection of the spleen and liver. Thus, we concluded that in a mouse model of melioidosis *B. pseudomallei* preferentially colonizes the stomach following oral inoculation, and that the chronically colonized GI tract likely serves as a reservoir for dissemination of infection to extra-intestinal sites.

## Introduction

*Burkholderia pseudomallei* is a soil and water bacterium that infects humans and other mammals in southeast Asia, northern Australia, Brazil, and other parts of the world [1], [2], [3]. Infection with *B. pseudomallei* can produce either acute, septicemic infections or chronic disseminated infections with long latency periods [4], [5], [6], [7]. Melioidosis is a particularly dangerous disease in humans because of the rapidity with which *B. pseudomallei* can cause disseminated infection and sepsis and because the organism displays high levels of intrinsic antibiotic resistance [7], [8]. These features, plus the fact that the organism is highly persistent in the environment, have caused *B. pseudomallei* to be classified as a category B select agent by the United States Centers for Disease Control and Prevention [9].

Infection with *B. pseudomallei* typically develops following exposure to bacteria in soil or water, though in 20–76% of cases the initial source of exposure remains unknown [10], [11], [12], [13], [14]. Patients infected with *B. pseudomallei* may remain asymptomatic for extended periods of time, in some cases for up to six decades following the original exposure to the organism [4], [5], [10], [15]. While acute melioidosis has been relatively well-studied, much less is known about how chronic disease develops or where the bacterium persists during long periods of asymptomatic infection.

*B. pseudomallei* is well-suited for survival in the soil and moist environments. For example, *B. pseudomallei* has been reported to survive completely without nutrients in distilled water for up to 2 decades [16], 17,18. Environmental surveys have shown that *B. pseudomallei* can be isolated from surface water over a wide pH range from 2–9 [19], [20]. Studies have also shown that chlorine-treated water does not effectively kill the *B. pseudomallei* organism [21], [22]. Moreover, *B. pseudomallei* can survive in feces for up to 27 days and in urine for up to 17 days [23].

Early studies of the pathogenesis of *B. pseudomallei* infection, carried out nearly a century ago using a number of different animal challenge models, provided convincing evidence of susceptibility to oral infection [23], [24], [25], [26]. For example, melioidosis could be induced by feeding *B. pseudomallei*-contaminated vegetables to monkeys, guinea pigs, rabbits, and black rats [23], [25], [26]. Melioidosis was also reported in a dog and a pig that ate meat contaminated with *B. pseudomallei*, and contaminated drinking water was responsible for two outbreaks on pig farms [27], [28], [29], [30].

In humans, *B. pseudomallei* has been isolated from gastric fluids, intestinal contents and from feces of melioidosis patients [26], [31], [32]. Human infection has been attributed to ingestion of contaminated lake or pond water, and contaminated drinking water was blamed for two melioidosis outbreaks in Australia [33], [34], [35]. Ulcers have been observed in the stomach, small intestine and colon of human melioidosis patients, and infants have developed melioidosis following consumption of culture positive breast milk [6], [36], [37], [38], [39]. Moreover, *B. pseudomallei* has been isolated from 26% of drinking water sources in Thailand and Australia [40], [41], [42], [43]. Therefore, there is mounting evidence that *B. pseudomallei* infection may be contracted orally in humans and that enteric infection may be more common than previously appreciated.

The goal of the current study was to determine where *B. pseudomallei* resided in mice during the chronic, subclinical phase of infection and to help discern how the organism disseminated to the spleen and liver during chronic infection. To address these questions, we initially developed a mouse model of chronic *B. pseudomallei* infection, using mucosal low-dose challenge studies in several different strains of mice. Using selective media and sensitive culture and detection techniques, we next investigated potential sites of bacterial persistence during the chronic infection phase of infection in mice.

Here we present evidence that *B. pseudomallei* can readily establish persistent GI infection in mice following very low-dose oral or intranasal inoculation. Remarkably, *B. pseudomallei* rapidly and persistently infected the entire GI tract following inoculation and appeared to preferentially colonize the gastric mucosa, without causing any discernable GI pathology or clinical signs. These findings suggest a possible explanation for maintenance of asymptomatic *B. pseudomallei* infection in humans, but also raise a number of new questions as to how gastric infection is maintained. This new animal model of chronic *B. pseudomallei* infection should prove useful in investigating host and pathogen factors that regulate GI colonization and shedding and for assessing new treatment modalities for elimination of infection.

## Results

### Persistent GI colonization develops following low-dose oral inoculation with *B. pseudomallei*, but not *B. thailandensis*

Previous studies have demonstrated infection with *B. pseudomallei* following acute, high-dose oral inoculation, but effects of oral inoculation on development of chronic infection have not been previously assessed [23], [24], [25], [44], [45]. To investigate the ability of *B. pseudomallei* to establish chronic enteric infection, we first determined the minimal oral challenge dose of *B. pseudomallei* strain 1026b required to cause persistent GI colonization. To ensure that the environmental survival traits of *B. pseudomallei* were not responsible for enteric persistence, oral challenge studies were also performed with *B. thailandensis* E264, a closely related but avirulent environmental bacterium [46], [47], [48]. The selective medium used to culture *B. pseudomallei* and *B. thailandensis* from GI organs in this study was Ashdown's medium (ASH) [49] supplemented with norfloxacin, ampicillin and polymyxin B (NAP-A). Preliminary studies demonstrated that growth of 25 clinical and soil *B. pseudomallei* strains, and 3 *B. thailandensis* strains, on NAP-A medium was equivalent when compared to growth on ASH, or LB agar media (Goodyear A., et al; manuscript in preparation). The additional antibiotics in NAP-A medium provided increased selectivity against enteric microflora of multiple mouse strains (BALB/c, C57BL/6, ICR) as compared to ASH (Goodyear A., et al; manuscript in preparation).

BALB/c mice (n = 9–10 per group) were inoculated with *B. pseudomallei* or *B. thailandensis* orally and quantitative cultures were done on GI organs 8 weeks after inoculation. Any animal where *B. pseudomallei* or *B. thailandensis* could be recovered from the stomach, small intestine (SI), cecum or colon on day 60 after inoculation was considered to be persistently infected. Oral inoculation with 4×10^5^ and 4×10^4^ CFU *B. pseudomallei* yielded similar results, with 89% and 78% of mice becoming chronically infected, respectively. Challenge with 4×10^3^ CFU *B. pseudomallei* resulted in a 44% rate of chronic infection, while inoculation with 2.5×10^2^ CFU *B. pseudomallei* resulted in a 10% chronic infection rate (**Table 1**). The infectious dose needed to persistently colonize 50% of mice (ID_50_) following oral *B. pseudomallei* infection was determined to be 1.5×10^4^ CFU according to the Reed-Muench method (Table 1). Therefore, for most experiments done in this study, an oral challenge dose of 5×10^5^ CFU *B. pseudomallei* strain 1026b was used, as this dose reliably caused persistent GI infection without triggering acutely fatal disease.

**Table 1:** Persistent gastrointestinal colonization following oral infection.

*B. pseudomallei*		*B. thailandensis*	
Dose (CFU)	% GI Infection	Dose (CFU)	% GI Infection
3.9×10^5^	89% (8/9)	2.8×10^10^	50% (5/10)
4.2×10^4^	78% (7/9)	2.6×10^9^	44% (4/9)
3.8×10^3^	44% (4/9)	2.2×10^8^	20% (2/10)
2.5×10^2^	10% (1/10)		
ID_50_ a = 1.5×10^4^ CFU		ID_50_ a = 1.3×10^10^ CFU	

a ID_50_ values calculated according to the Reed-Muench method [101].

In contrast to oral *B. pseudomallei* infection, very high doses of *B. thailandensis* were required to cause persistent GI colonization. Organ plating performed at day 60 following oral infection with *B. thailandensis* revealed that 50% of mice were infected with 2.8×10^10^ CFU, 44% of mice infected with 2.6×10^9^, and 20% of mice infected with 2.2×10^8^ CFU were persistently infected (Table 1). The ID_50_ following oral *B. thailandensis* infection determined by the Reed Muench method was 1.3×10^10^ CFU. This is approximately a 6 log_10_ increase from the ID_50_ observed for *B. pseudomallei* (Table 1). Additionally, fecal shedding titers determined at earlier time points were also reduced following oral inoculation of mice with 2.8×10^10^ CFU *B. thailandensis* as compared to oral infection with 5×10^5^ CFU *B. pseudomallei*. For example, a statistical trend was observed at day 3 (*p* = 0.07) while titers were significantly reduced in *B. thailandensis* inoculated mice at day 14 (*p*<0.05), 35 (*p*<0.01) and 56 (*p*<0.01) (data not shown). These studies demonstrated therefore that environmental survival traits alone were not sufficient to explain gastrointestinal persistence observed following *B. pseudomallei* infection.

In addition, we investigated the ability of oral challenge with *B. pseudomallei* to elicit acutely lethal infection by determining LD_50_ values according to the Reed-Muench method. LD_50_ values for acute disease following oral challenge (ie, mice euthanized on or before day 7) in BALB/c, C57BL/6 and 129S6/SvEv mice (n = 6–10 per dose), were 1.04×10^7^ CFU, 7.1×10^6^ CFU, and 1.9×10^3^ CFU respectively. From these studies, we observed mouse strain-specific differences in susceptibility to acute, lethal infection following oral inoculation. For instance, 129S6/SvEv mice were highly susceptible to oral infection, similar to results following respiratory infection [50]. In contrast to previous reports demonstrating that C57BL/6 mice are more resistant than BALB/c mice following intranasal, subcutaneous, or oral infection; these two mouse strains were equally susceptible to oral infection in this study [45], [51], [52]. This result may be unique to the combination of oral infection with *B. pseudomallei* strain 1026b, as similar results were observed in a study of acute enteric infection performed with *B. pseudomallei* strain 1026b [44]. Following oral infection of BALB/c mice (n = 30) with *B. thailandensis* strain E264 only one death was observed 25 days after infection with 2.6×10^9^ CFU, while no deaths were observed following oral infection with 2.2×10^8^ or 2.2×10^10^ CFU. Thus, oral inoculation with *B. pseudomallei* strain 1026b, but not *B. thailandensis*, reliably produced either chronic or acute infection in mice, depending on the challenge dose delivered.

### *B. pseudomallei* is present in all GI organs following oral inoculation

**Figure 1:**
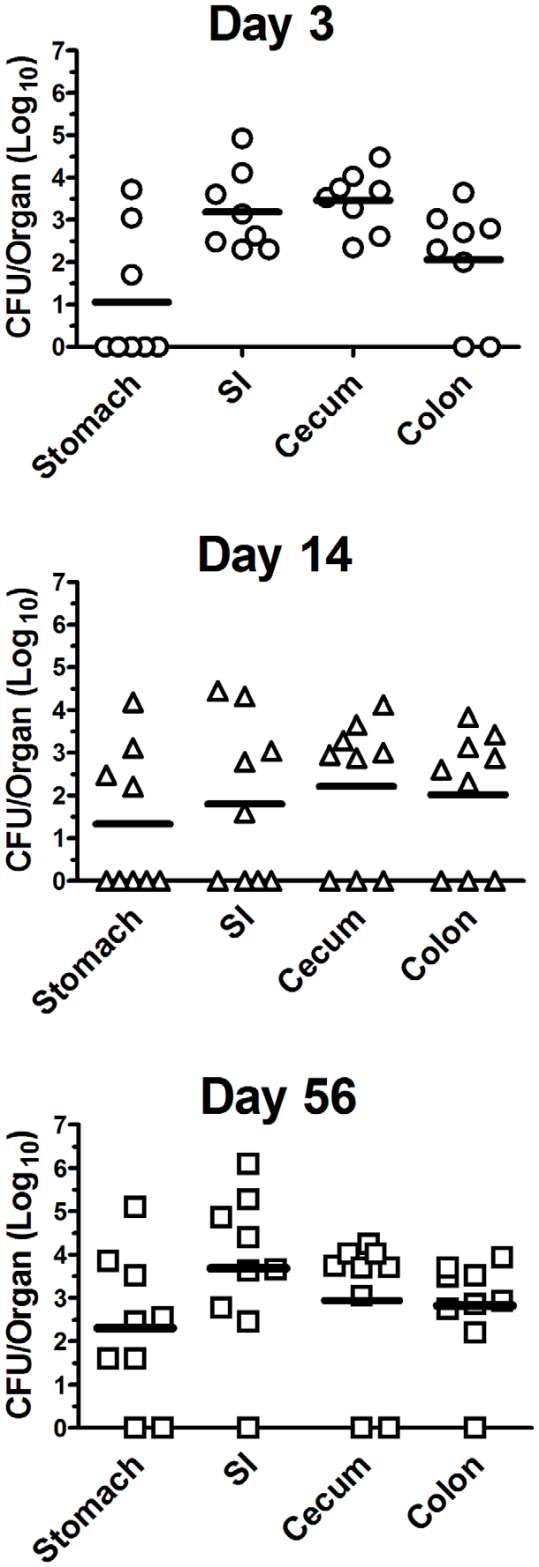
Gastrointestinal bacterial burden following oral *B. pseudomallei* challenge. BALB/c mice (n = 8–9) were inoculated orally with 5×10^5^ CFU *B. pseudomallei* strain 1026b. On day 3, 14 and 56 after inoculation, mice were euthanized and organs were processed for determination of bacterial burden as described in Materials and Methods. Data are presented as individual log_10_ CFU/organ values with bars representing the mean titer for each organ. The limit of detection was 20 CFU/organ. Data were pooled from two independent experiments.

Using the low-dose oral challenge model, we next investigated whether there were differences in the location of persistent *B. pseudomallei* infection amongst GI organs. BALB/c mice (n = 8–9 per group) were inoculated orally with 5×10^5^ CFU *B. pseudomallei* and bacterial burdens in various locations in the GI tract were determined on days 3, 14 and 56 post-challenge. We observed that persistent infection could be detected in most mice at all levels of the GI tract examined, from the stomach through the colon (**Figure 1**). Bacteria could be recovered more frequently from the small intestine, cecum, and colon and at higher titers than from the stomach.

In mice inoculated orally with *Salmonella*, *Yersinia enterocolitica*, *or Shigella dysenteriae*, the gallbladder and mesenteric lymph nodes are often infected [53], [54], [55], [56], [57], [58]. However, in mice challenged orally with *B. pseudomallei*, we found that the mesenteric lymph nodes or the gall bladder were rarely infected, and if positive were infected only at a very low level. For example, mesenteric lymph node infection was found in only 8 of 36 mice, with a mean titer of 9 CFU/organ (data not shown). Likewise, the gall bladder was infected in only 2 of 26 mice, with a mean titer of 3 CFU/organ (data not shown).

**Figure 2:**
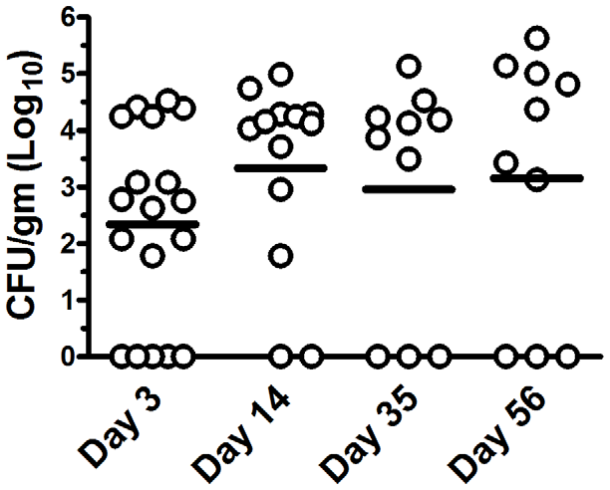
*B. pseudomallei* is persistently shed in the feces following oral inoculation. BALB/c mice (n = 10–18 animals per group) were inoculated orally with 5×10^5^ CFU. On days 3, 14, 35 and 56 after inoculation, feces were collected and processed for determination of bacterial burden as described in Materials and Methods. Data are presented as individual log_10_ CFU/gram values with bars representing the mean titer at each time point. The limit of detection was 10–60 CFU/gram, depending on the number of fecal pellets collected from each mouse. Data was pooled from two independent experiments.

Given the ability of *B. pseudomallei* to persistently colonize the GI tract, we next asked whether infected mice also shed the organism in their feces. Therefore, we examined fecal shedding of *B. pseudomallei* from orally-inoculated mice (5×10^5^ CFU) over a 60 day period (**Figure 2**). We found that fecal shedding of *B. pseudomallei* could be detected beginning as early as 24 hours following oral inoculation (data not shown). Remarkably, the level of fecal shedding remained relatively constant over the next 60 days of observation. The concentration of *B. pseudomallei* in feces of persistently infected mice (range = 10^2^–10^5^ CFU/gm feces, average = 10^3^ CFU/gm feces). It should also be noted that persistently infected mice showed no outward signs of GI infection, maintaining normal body weight and fecal pellet consistency.

### GI infection develops following oral inoculation with multiple different *B. pseudomallei* strains

**Figure 3:**
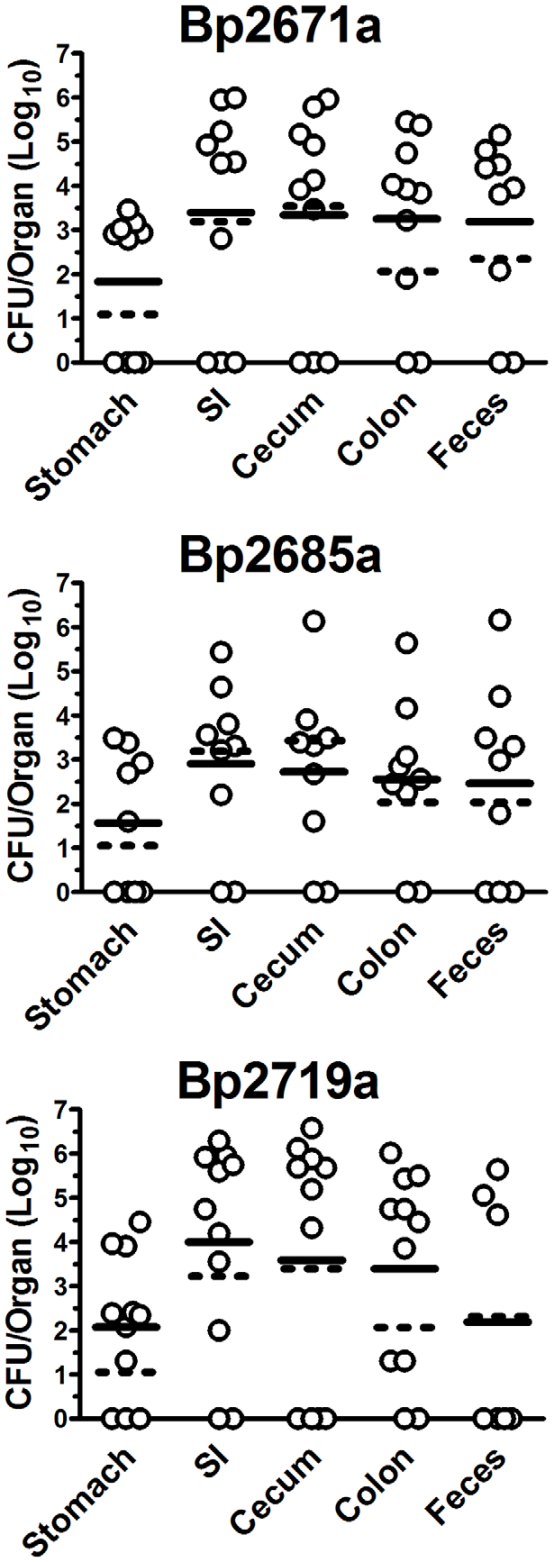
GI colonization occurs following oral inoculation with multiple *B. pseudomallei* strains. BALB/c mice (n = 9–11 animals evaluated per bacterial strain) were inoculated orally with Bp2671a (3.6×10^5^ CFU); Bp2685a (2.9×10^5^ CFU); or Bp2719a (3.5×10^5^ CFU). At day 3 after infection, organs and feces were processed for determination of bacterial burden. Data are presented as individual values with solid bars representing the mean log_10_ titer. Organ bacterial burdens are expressed as log_10_ CFU/organ, and feces titers are graphed as log_10_ CFU/gram of feces. Dashed bars represent the mean log_10_ titers from day 3 Bp1026b bacterial burden determination (Reproduced from Figure 1 for reference). Data were pooled from 2 independent experiments. The limit of detection was 20 CFU/organ, and 10–60 CFU/gram of feces, depending on the number of fecal pellets collected from each mouse.

The preceding experiments done with *B. pseudomallei* strain 1026b demonstrated a marked propensity to colonize the GI tract and establish persistent infection following oral inoculation. To determine whether the ability to establish GI infection was a general property of *B. pseudomallei*, or instead reflected a strain-specific phenomenon, we also subjected mice to oral challenge with 3 additional strains (Bp2671a, Bp2685a, Bp2791a) of *B. pseudomallei*. These 3 strains were randomly selected from a panel of 22 clinical *B. pseudomallei* isolates kindly provided by Dr. S. Peacock (now at the University of Cambridge). In initial experiments, we observed that oral challenge with a challenge dose of ∼5×10^5^ CFU with the 3 new strains of *B. pseudomallei* produced a high percentage of acutely lethal infections, suggesting that these 3 strains were each more virulent than the 1026b strain (data not shown). Due to the increased acute lethality of these 3 new strains, colonization of GI organs at day 3 post infection was used to compare infection with 1026b (see Figure 1) and the 3 new strains. BALB/c mice (n = 8–10 per strain) were challenged orally with each of the 3 additional *B. pseudomallei* strains (Bp2671a = 3.6×10^5^ CFU; Bp2685a = 2.9×10^5^ CFU; Bp2719a = 3.5×10^5^ CFU), and the organ bacterial burden was determined on day 3. We did not observe statistically significant differences in GI bacterial burdens in mice infected with strain 1026b compared to the 3 new clinical isolates (**Figure 3**).

To determine whether lower oral challenge doses could elicit chronic GI infection with the 3 clinical *B. pseudomallei* strains, BALB/c mice (n = 3–5 per group) were inoculated with approximately 5×10^4^ CFU of each of the 3 new *B. pseudomallei* strains (Bp2671a = 2.0×10^4^ CFU; Bp2685a = 4.8×10^4^ CFU; Bp2719a = 2.8×10^4^ CFU). Even a one log reduction in challenge dose still resulted in rapid lethality with a 58% mortality rate by day 14 post-challenge (data not shown). Determination of bacterial burden in feces of surviving mice that were challenged orally with the 3 new clinical strains of *B. pseudomallei* revealed that 6 of 8 mice had GI colonization on day 7, while 3 of 5 mice were colonized on day 14, although the two surviving mice had cleared the GI infection by day 56 (data not shown). Strain Bp2719a was found to be especially virulent and p.o. infection with as few as 1.8×10^3^ CFU resulted in 100% lethality 5 days after infection (data not shown). Thus, acute challenge studies revealed that all 4 strains of *B. pseudomallei* were similar in their ability to colonize the GI tract following oral inoculation. However, the three low-passage clinical isolates also appeared to be more virulent *in vivo* than *B. pseudomallei* 1026b, as reflected by more rapid spread and dissemination following oral challenge (See below).

### Dissemination to systemic organs following oral *B. pseudomallei* inoculation

**Figure 4:**
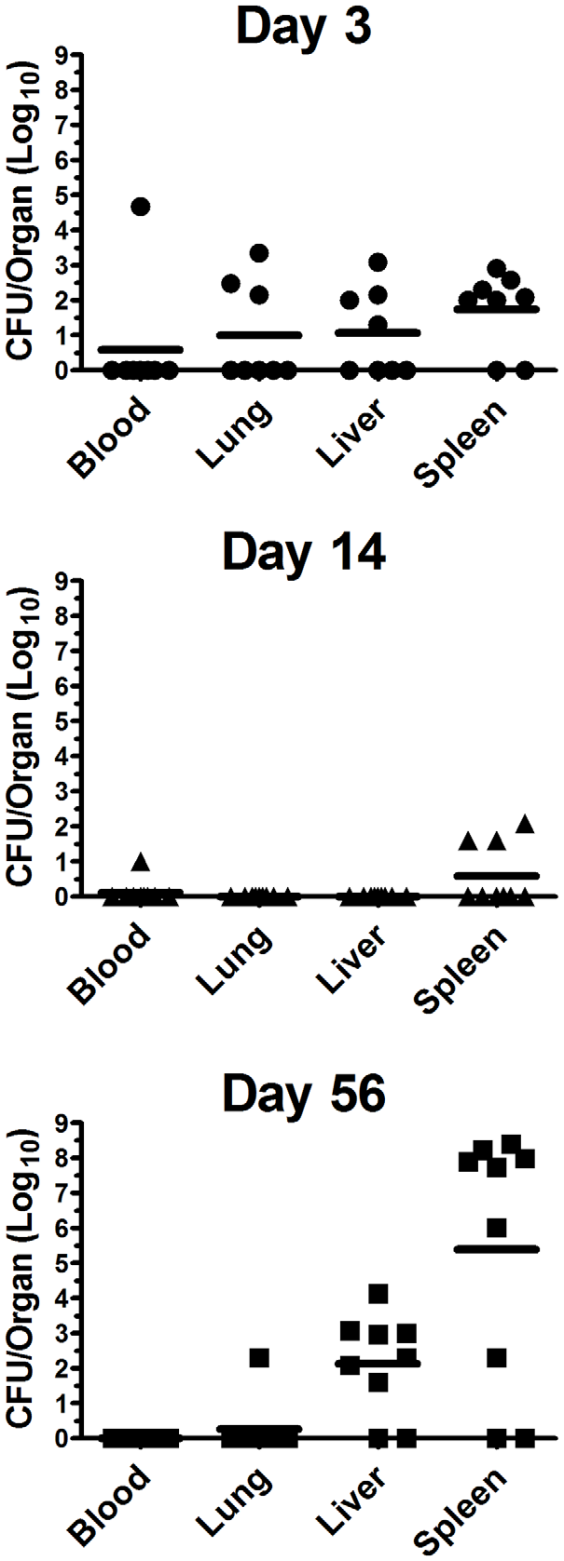
Bacterial dissemination to systemic organs following oral inoculation with *B. pseudomallei*. BALB/c mice (n = 8–10 animals per time point) were inoculated orally with 5×10^5^ CFU Bp1026b. On day 3, 14 and 56 after inoculation mice were euthanized and organs were processed for determination of bacterial burden. Data are presented as mean ± SEM log_10_ values. Lung, liver and spleen titers are graphed as CFU/organ, and blood is graphed as CFU/ml. The limit of detection was 20 CFU/organ, and 10 CFU/ml for blood. Data were pooled from two independent experiments.

The liver and the spleen are two of the most frequently affected visceral organs in humans with melioidosis [3], [10], [59], [60], [61]. We and others have also observed that in mice that survive high-dose intranasal challenge with *B. pseudomallei*, death due to disseminated infection to the liver and spleen often develops over a 30–90 day period [3], [62], [63], [64]. Therefore, we next investigated whether disseminated infection to the liver and spleen could develop following oral inoculation with *B. pseudomallei*. To address this question, bacterial burdens in the blood, lung, liver, spleen were determined 3, 14 and 56 days after low-dose oral challenge of BALB/c mice (n = 8–10) with 5×10^5^ CFU *B. pseudomallei* 1026b. We observed that by day 56 post-challenge, nearly all orally-inoculated mice had *B. pseudomallei* lesions in the spleen and liver, with especially high bacterial burdens in the spleen (**Figure 4**). Thus, it appeared that *B. pseudomallei* could readily disseminate from the GI tract to the liver and spleen.

We next assessed whether the 3 low-passage *B. pseudomallei* strains could also disseminate to the liver and spleen following low-dose oral inoculation. Mice (n = 9–11 per challenge strain) were inoculated orally (∼5×10^5^ CFU) with each of the three strains and bacterial burdens were determined in blood, lung, liver, spleen three days after inoculation. Each of these three strains was able to cause infection in the blood, lung, liver, and spleen, with bacterial burdens statistically equivalent to those generated by oral inoculation with *B. pseudomallei* strain 1026b; although a trend towards increased burdens in the liver (*p* = 0.08) and spleen (*p* = 0.07) were observed following infection with strain Bp2719a as compared to Bp1026b, when analyzed by a two-tailed Student's t-test (**Figure S1**).

After receiving a low dose oral challenge (∼5×10^4^ CFU) with strain Bp2671a, Bp2685a or Bp2719a, 6 of 11 mice had grossly visible splenic lesions, while only 2 of 9 mice inoculated orally with Bp1026b (5×10^5^ CFU) had spleen lesions at day 60 post-challenge. Thus, it was apparent that different *B. pseudomallei* strains were capable of disseminating from the GI tract following oral inoculation.

### *B. pseudomallei* colonizes the stomach following oral infection

The above experiments demonstrated that *B. pseudomallei* persistently colonized all organs of the GI tract. However, we were interested to determine exactly where the infection was maintained in the GI tract, including the GI lumen, the GI mucosa, or submucosal tissues. To address this question, fluorescent *in situ* hybridization (FISH) was performed to localize *B. pseudomallei* within GI tissues. Preliminary FISH experiments performed with cultured bacteria and mixed fecal bacteria demonstrated that the Bpm427 and Bpm975 probes bound to *B. pseudomallei* strain 82 (Bp82) but not to *B. thailandensis* or endogenous fecal bacteria (data not shown). Additionally, when Bp82 was added into mixed fecal bacteria, the Eub338 signal from Bp82 was found to be dim as compared to the signal from fecal bacteria. Therefore, when the 6-FAM signal from the Eub338 probe was combined with the Cy3 signal from the Bpm427 and Bpm975 probes, *B. pseudomallei* typically appeared red in image overlays rather than yellow (data not shown). For bacterial localization studies, BALB/c mice were inoculated with ∼5×10^5^ CFU *B. pseudomallei* strain 1026b and mice were euthanized 56 days after infection. Tissues were fixed and hybridized with FISH probes as described in Materials and Methods. To distinguish *B. pseudomallei* staining from that of intestinal debris, tissues were observed at 1000× final magnification.

**Figure 5:**
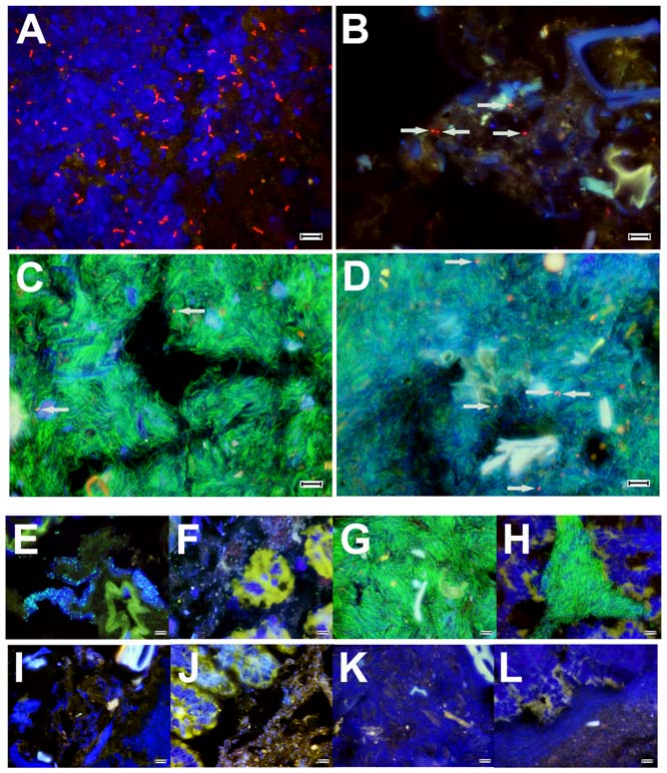
Localization of *B. pseudomallei* 1026b in gastrointestinal organs following oral infection. (A–D) Stomach (A), small intestine (B), cecum (C) and colon (D) tissues from mice infected orally with 5×10^5^ CFU *B. pseudomallei* strain 1026b were collected 56 days after infection. Organs were fixed in 10% NBF and embedded in paraffin prior to sectioning. FISH was performed on tissue sections as described in materials and methods. Tissue sections were counterstained with DAPI (blue) and observed at 1000× final magnification. Tissue sections were hybridized with a eubacterial probe (green), and two *B. pseudomallei* specific probes (red). (E–L) Control tissues from uninfected BALB/c mice were processed as described for *B. pseudomallei* infected tissues. To ensure *B. pseudomallei* probes did not cross react with enteric bacteria, stomach (E) small intestine (F), cecum (G), and colon (H) tissues from uninfected BALB/c mice were hybridized with a eubacterial probe (green), and both *B. pseudomallei* specific probes (red). To ensure the FISH procedure resulted in specific probe hybridization, stomach (I), small intestine (J) cecum (K) and colon (L) tissues from uninfected BALB/c mice were hybridized with an irrelevant probe (green). Arrows in B–D indicate the location of *B. pseudomallei*. In all images the scale bar represents 10 microns.

Remarkably, FISH staining revealed that the major site of persistent *B. pseudomallei* infection following oral inoculation was in the stomach (**Figure 5A**). Clusters of bacteria appeared to be primarily cell-associated in the stomach, and most were found on the surface of gastric mucosal cells within gastric pits, especially in the fundus and gastric antrum and pyloric regions of the stomach. In contrast, *B. pseudomallei* was much less numerous throughout the rest of the GI tract, including the small intestine, ileum, cecum, and large intestine and colon (**Fig. 5B–D**). In addition, bacteria in the intestine and colon were typically not associated with mucosal cells, but were instead found primarily within intestinal luminal contents. These findings suggested that *B. pseudomallei* existed in very different niches in the stomach versus the intestines, being presumably attached to the mucosa in the stomach and free in the lumen in the intestines.

**Figure 6:**
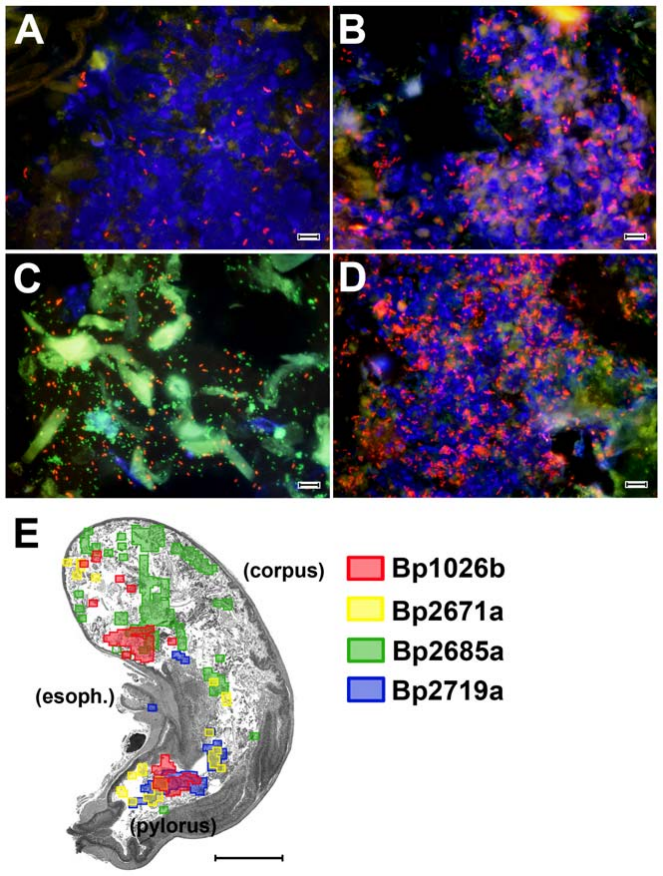
Gastric colonization following oral infection with different *B. pseudomallei* isolates. BALB/c mice were infected orally with *B. pseudomallei* strain Bp1026b (5×10^5^ CFU), Bp2671a (2.0×10^4^ CFU), Bp2685a (4.8×10^4^ CFU) or Bp2719a (2.8×10^4^ CFU). Stomach tissues were collected from Bp1026b mice 56 days after infection, Bp2671a mice 21 days after infection, Bp2685a mice 3 days after infection, and Bp2719a mice 4 days after infection. All tissues were fixed in 10% NBF and embedded in paraffin before sectioning. FISH was performed on stomach tissue sections from mice infected with Bp1026b (A), Bp2671a (B), Bp2685a (C) and Bp2719a (D) as described in materials and methods. Tissues were counterstained with DAPI (blue), and observed at 1000× final magnification. Tissue sections were hybridized with a eubacterial probe (green) and two *B. pseudomallei* specific probes (red). (E) The location of 1000× fields positive for *B. pseudomallei* was determined for each *B. pseudomallei* isolate. Positive 1000× fields are indicated by red (Bp1026b), yellow (Bp2671a), green (Bp2685a) or blue (Bp2719a) shaded outlines. Outlines were overlaid onto a representative stomach image created by combining images from a hematoxylin and eosin stained section. The esophagus (esoph.), body (corpus) and pylorus of the stomach are labeled for reference. In (A–D) the scale bar represents 10 microns, and in (E) the scale bar represents 2 mm.

Additional FISH studies revealed that all 4 *B. pseudomallei* isolates also colonized the stomach (**Figure 6**). BALB/c mice were inoculated orally with three additional isolates (Bp2671a = 2.0×10^4^ CFU; Bp2685a = 4.8×10^4^ CFU; Bp2719a = 2.8×10^4^ CFU). Organs were harvested from Bp2671a on day 21, from Bp2685a on day 3, and Bp2719a on day 4 after infection. In mice infected with Bp1026b, Bp2671a and Bp2719a, *B. pseudomallei* organisms were almost exclusively co-localized with tissue DAPI staining. In contrast, in gastric tissues of Bp2685a infected mice, the majority of *B. pseudomallei* organisms were identified primarily in ingesta in the lumen of the stomach. Infection with Bp1026b, Bp2671a and Bp2719a resulted in scattered but concentrated foci of infection, whereas strain Bp2685a colonization was evenly distributed over larger areas of the stomach. Similar to Bp1026b, all 3 additional *B. pseudomallei* isolates were identified at low levels in the ingesta of the small intestine and cecum as well as the fecal material in the colon (**Figures S2, S3, S4**). Therefore, regardless of the *B. pseudomallei* strain used, or the time points that organs were examined, the stomach was the most heavily colonized organ.

To determine which regions of the stomach were colonized following oral infection, a mosaic of positive 1000× fields from each stomach was prepared. Positive 1000× fields were then overlaid over a representative stomach image (**Fig. 6E**). This analysis revealed that *B. pseudomallei* was located primarily in the pylorus and body of the stomach, and in one case was identified in the esophagus (Fig. 6E). Similar to *Helicobacter pylori* infection *B. pseudomallei* colonized the pylorus, although *B. pseudomallei* also colonized the body of the stomach [65]. Individual localization mosaic overlays generated using DAPI composite images from each stomach are shown in **Figure S5**.

To guard against the possibility of non-specific binding of *B. pseudomallei* probes to enteric bacteria, GI tissues from uninfected BALB/c mice were hybridized with the Bpm427, Bpm975 and Eub338 probes. These experiments demonstrated that the *B. pseudomallei* probes did not cross-react with enteric bacteria, while at the same time the eubacterial probe did bind to enteric bacteria (Fig. 5E–H). Tissues from uninfected mice were also hybridized with the irrelevant Non338 probe to ensure that binding of the FISH probes was sequence specific. While bacterial DAPI signal was observed in all GI sections, no signal was observed from the Non338 probe, demonstrating that the FISH procedure resulted in sequence specific probe binding (Fig. 5I–L).

Focal clusters of intense *B. pseudomallei* infection were only identified in the stomach (Fig. 5, Fig. S2Fig S2, S3, S4). Although rare, submucosal localization of *B. pseudomallei* within the ileum of the small intestine was consistently noted in tissues from mice infected with each of the 4 *B. pseudomallei* strains tested. In contrast, *B. pseudomallei* could not be localized by FISH in cecum or colon tissue from mice inoculated with either of the 4 *B. pseudomallei* strains (data not shown). These results suggested therefore that following oral inoculation, *B. pseudomallei* established persistent infection of the stomach, which resulted in downstream shedding of bacteria into the small and large intestines, which in contrast to the stomach appeared not to be colonized by *B. pseudomallei*.

### Mice lack gastrointestinal pathology following enteric *B. pseudomallei* infection

**Figure 7:**
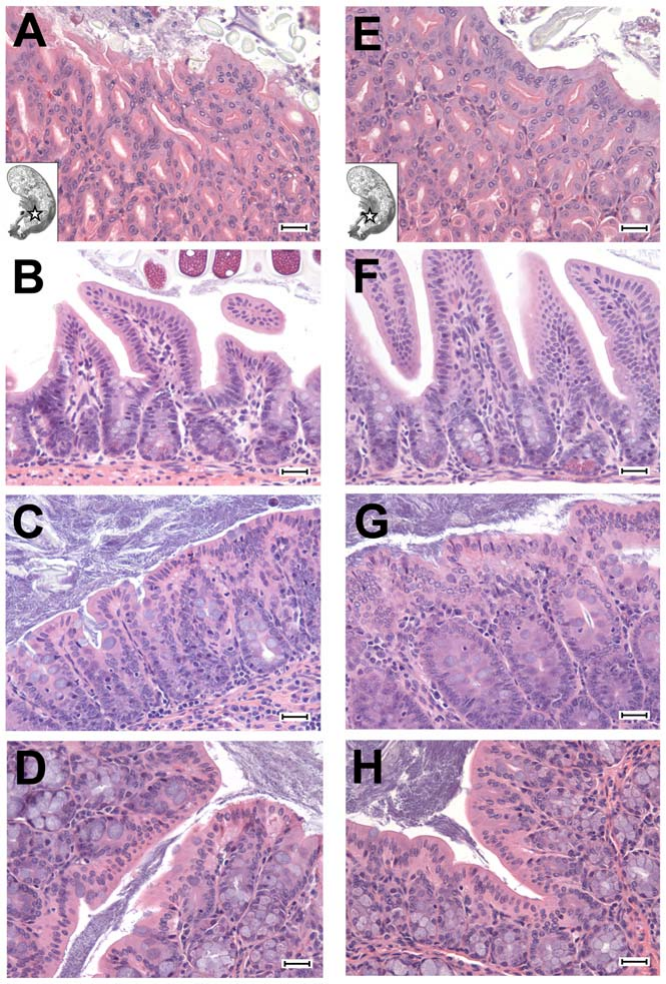
Histology in gastrointestinal organs following oral *B. pseudomallei* infection. BALB/c mice were in inoculated with 5×10^5^ CFU *B. pseudomallei* 1026b. Mice were euthanized 56 days after infection and tissues were processed and stained with hematoxylin and eosin as described in methods. Tissues were also collected from uninfected BALB/c mice as control tissues. Stomach (A), small intestine (B), cecum (C) and colon (D) images from uninfected BALB/c mice are shown in the left column. Stomach (E), small intestine (F), cecum (G) and colon (H) images from *B. pseudomallei* infected mice are shown in the right hand column. For stomach images (A, E) the location of each H+E image within the stomach is indicated by the star on the stomach outline shown in the bottom left corner of each image. All small intestine images are from the ileum, and the colon images are from the proximal colon. Images were captured at 400× final magnification, and the scale bar on all images represents 25 microns.

Studies were done next to determine if *B. pseudomallei* enteric infection was associated with GI lesions. Sections from tissues used for FISH analysis were analyzed for histopathologic changes. BALB/c mice (n = 32) were inoculated orally with each of 4 different *B. pseudomallei* strains (Bp1026b = 5×10^5^ CFU; Bp2671a = 2.0×10^4^ CFU; Bp2685a = 4.8×10^4^ CFU; Bp2719a = 2.8×10^4^ CFU). Tissues were analyzed from *B. pseudomallei* infected mice at multiple time points following infection (Bp1026b days 2, 3, 14, and 56; Bp2671a days 4, 8, and 21; Bp2685a days 2 and 3; Bp2719a days 4, 8, and 11). Fecal titers of *B. pseudomallei* were determined prior to euthanasia to assure that the GI tracts of mice were heavily colonized (up to 10^6^ CFU/gm; data not shown). Despite the presence of large number of bacteria associated with the gastric mucosa as demonstrated by FISH, gastric lesions were not observed in the infected animals (**Figure 7** **, Figure S6**). Moreover, the cecum and colon tissues of infected mice were also free of lesions. The only lesions noted (in 2 of 32 mice examined) consisted of mild neutrophil and macrophage infiltrates in the ileum. In one of these infected animals there was a noticeable vasculitis and necrosis in the serosa associated with neutrophil and macrophage infiltration, while necrosis was not observed in the other animal examined (data not shown).

### Ability of *B. pseudomallei* to infect the GI tract after challenge by non-oral routes of inoculation

The preceding studies indicated that *B. pseudomallei* readily colonized the GI tract following oral inoculation. We therefore examined whether other routes of inoculation could also produce chronic enteric infection. Fecal shedding following challenge with *B. pseudomallei* strain 1026b was used to survey mice for evidence of enteric colonization following intranasal (i.n.), intraperitoneal (i.p.), or subcutaneous (s.c.) inoculation.

**Figure 8:**
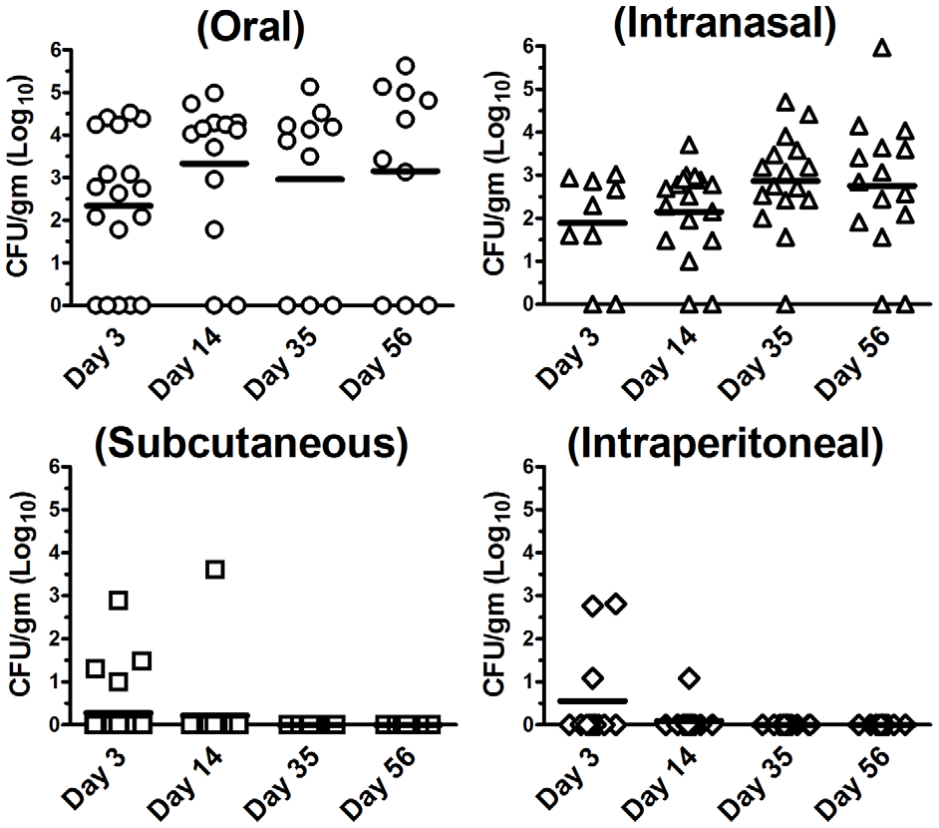
*B. pseudomallei* is persistently shed in feces following i.n. inoculation, but not after s.c. or i.p. inoculation. BALB/c mice were inoculated with *B. pseudomallei* using the following route and dose combinations: Oral inoculation (5×10^5^ CFU) (n = 10–18 animals), i.n. inoculation (∼500 CFU) (n = 9–17 animals), s.c. inoculation (5×10^4^ CFU–5×10^7^ CFU) (n = 11–24 animals), or i.p. inoculation (10^6^ CFU–10^8^ CFU) (n = 11–12 animals). On day 3, 14, 35 and 56 after infection, fecal pellets were collected and processed for determination of bacterial burden. Data are graphed as individual log_10_ CFU/gram values with bars representing the mean value for each time point. Oral fecal shedding data from figure 2 are reproduced in this figure for reference. Data were pooled from 2–6 experiments per infection route.

Intranasal inoculation resulted in high levels of enteric infection, whereas inoculation by the s.c. or i.p. routes produced very different results (**Figure 8**). For example, none of the mice inoculated by the s.c. or i.p. routes developed persistent enteric infection. In contrast, following i.n. inoculation of BALB/c mice with a very low bacterial challenge dose (approximately 500 CFU per mouse), 13 of 15 mice (87%) developed persistent enteric infection. Interestingly, i.n. inoculation also resulted in a higher percentage of mice with fecal shedding compared to mice subjected to oral inoculation (87% versus 70%). At present, the route by which *B. pseudomallei* colonizes the GI tract following i.n. inoculation has not been conclusively determined.

**Figure 9:**
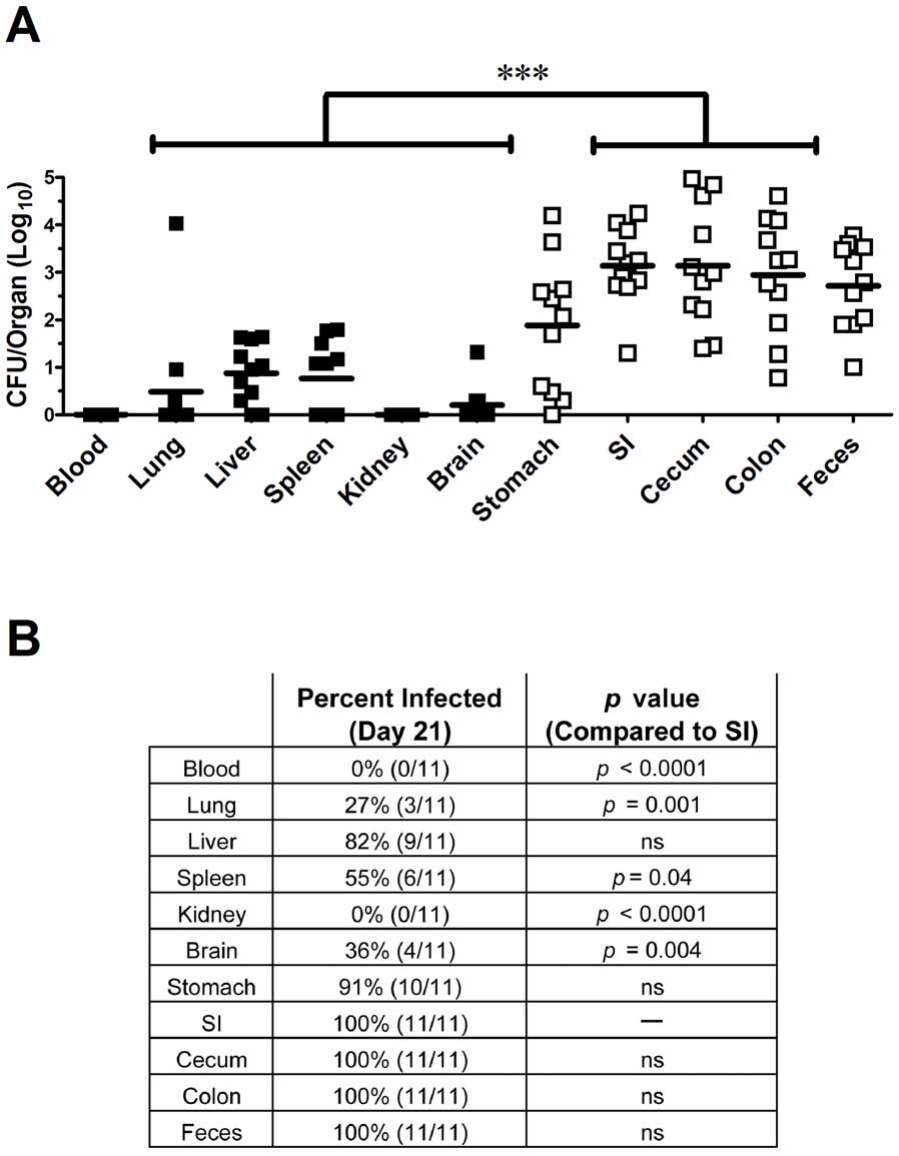
GI organs colonized more heavily and more frequently than extra-intestinal organs after i.n. challenge. BALB/c mice (n = 11 animals per group) were infected via the i.n. route with ∼500 CFU Bp1026b. After 21 days, blood, organs and feces were processed for determination of bacterial burden. (A) Bacterial burden in each organ following low-dose i.n. infection. Data are graphed as individual values with bars representing the mean log_10_ titer. Organ titers are plotted as log_10_ CFU/organ, blood as log_10_ CFU/ml, and feces as log_10_ CFU/gram. Statistical differences between organs were determined using a one-way ANOVA followed by a Tukey's multiple means test. (*** = *p*<0.001). (B) The percentage of mice with positive *B. pseudomallei* cultures from each organ was determined. Statistical differences between percentages in the intestine versus other organs were determined using a two-tailed Fisher's exact test. The limit of detection was 1 CFU/organ, 10 CFU/ml for blood, and 10–60 CFU/gram for feces, depending on the number of fecal pellets collected from each mouse. Both figures were generated by pooling data from 3 independent experiments. ns = not significant.

The preceding experiments led us to hypothesize that the GI tract was the primary site of bacterial persistence in mice inoculated by the oral or i.n. routes. To test this hypothesis, we inoculated BALB/c mice (n = 11) with a low dose (∼500 CFU) i.n. challenge with *B. pseudomallei* 1026b. Mice were euthanized on day 21, when all mice were clinically asymptomatic. Bacterial burdens were determined in blood, lung, liver, spleen, kidney, brain, stomach, small intestine, cecum, colon, and feces, using very sensitive culture techniques. To increase the sensitivity of bacterial detection, the entire organ homogenate from each organ was plated, using large agar plates (limit of detection of 1 CFU/organ). When bacterial burdens in all organs were compared, significant differences in bacterial burden were observed between the GI tract and extra-intestinal organs (lung, liver, spleen, brain and kidney) (**Figure 9**). For example, the small intestine, cecum, and colon each had significantly higher bacterial counts than the lung, liver, brain, kidney, or spleen (*p*<0.001). Differences were also observed between the stomach and extra-intestinal organs, although to a lesser degree (*p*<0.05). However, bacterial counts in the lung, liver, spleen, kidney or brain were not significantly different from one another, nor were significant differences observed between bacterial counts in various regions of the GI tract, including the stomach, small intestine, cecum, and colon. The GI tract was also much more likely to be infected compared to extra-intestinal organs. For example, the GI tract was colonized significantly more often than the lung, brain (*p*<0.01), spleen (*p*<0.05), or kidney (*p*<0.001) (Figure 9).

### Bacterial dissemination occurs more rapidly following oral inoculation than after s.c. inoculation

Currently, humans are thought to acquire *B. pseudomallei* infection primarily via accidental cutaneous inoculation with the organism [66]. However, it is also possible that other routes of infection, including oral exposure, may also lead to the development of chronic melioidosis and later disseminated infection. To compare the relative frequency with which chronic disease developed following cutaneous versus oral inoculation with *B. pseudomallei* strain 1026b, mice were inoculated s.c. or orally with an infectious dose of 5×10^4^ CFU (0.002×LD_50_ for s.c. and 0.004×LD_50_ for oral). The mice were euthanized on day 56 post infection and bacterial concentrations were determined in blood, lung, liver, spleen, kidney, brain, stomach, small intestine, cecum, colon and feces. None of the mice inoculated by the s.c. route had detectable bacteria in any organ cultured (**Table 2**). In contrast, 67% of mice inoculated orally had evidence of systemic, extra-intestinal infection. For example, *B. pseudomallei* was isolated from the liver of 6 of 9 mice (*p*<0.01) and from the spleen of 5 of 9 mice (*p*<0.05) inoculated orally. Similar results were also obtained at day 56 following low dose i.n. inoculation, which also produced persistent GI colonization (**Figure 7**). Compared to s.c. inoculation with 5×10^4^ CFU *B. pseudomallei*, i.n. inoculation with ∼500 CFU *B. pseudomallei* resulted in liver infection in 6 of 7 mice (*p*<0.001) and splenic infection in 4 of 7 mice (*p*<0.05) (data not shown).

**Table 2:** Systemic and gastrointestinal colonization following oral or subcutaneous infection.

	Challenge Dose = 5×10^4^ CFU		
Tissue	Oral	Subcutaneous	*p* value^a^
**Blood**	44% (4/9)	0% (0/10)	*p* = 0.03
**Lung**	56% (5/9)	0% (0/10)	*p* = 0.01
**Liver**	67% (6/9)	0% (0/10)	*p* = 0.003
**Spleen**	56% (5/9)	0% (0/10)	*p* = 0.01
**Stomach**	56% (5/9)	0% (0/10)	*p* = 0.01
**SI**	78% (7/9)	0% (0/10)	*p* = 0.0007
**Cecum**	78% (7/9)	0% (0/10)	*p* = 0.0007
**Colon**	78% (7/9)	0% (0/10)	*p* = 0.0007
**Feces**	60% (3/5)	0% (0/10)	*p* = 0.02

a Statistical differences between oral and subcutaneous infection were determined by Fisher's exact test.

Mice inoculated orally were also much more likely to die from chronic *B. pseudomallei* infection than mice inoculated subcutaneously. While the day 56 LD_50_ for s.c. inoculation was 4.9×10^6^ CFU, the day 56 LD_50_ for oral inoculation was only 5.9×10^4^ CFU. These results were consistent therefore with the idea that bacterial dissemination and chronic disease developed much more readily following oral inoculation and establishment of persistent GI colonization with *B. pseudomallei* strain 1026b.

As noted above, systemic infection was not observed in any of 10 mice subjected to s.c. challenge dose with 5×10^4^ *B. pseudomallei* strain 1026b. However, in 5 mice subjected to much higher challenge doses of *B. pseudomallei* by the s.c. route (dose range: 6.6×10^6^–2.6×10^7^ CFU), systemic infection did develop in 4 of 5 animals. Notably, all of these mice also developed persistent cutaneous lesions at the s.c. injection site. When these injection site lesions were cultured, we found very high bacterial titers, with an average titer of 3×10^7^ CFU *B. pseudomallei* per lesion. Therefore, these results suggest that efficient dissemination of *B. pseudomallei* to other organs may require either a nidus of high-level infection (eg, cutaneous lesions) or persistent, lower-level infection of the stomach.

## Discussion

Most studies have indicated that melioidosis in humans results primarily from inhalation or cutaneous inoculation of *B. pseudomallei* from the environment [66]. However, melioidosis also develops in 20–76% of patients with no known exposure to the organism [10], [11], [12], [13], [14]. Thus, it is possible that an alternative route of infection, such as oral inoculation, may be responsible for a number of melioidosis cases. In fact, compelling data from studies done at the turn of the century indicated that *B. pseudomallei* was in fact quite infectious in a variety of animal species following ingestion of the organism [23], [24], [25], [26]. Moreover, recent epidemiological studies also indicate that oral infection with *B. pseudomallei* may be possible [31], [37]. Recent studies in mice have demonstrated that oral infection with *B. pseudomallei* can cause acute disease, and results in antibody production and systemic infection [44], [45].

In our present study, we demonstrated that low dose oral inoculation produced persistent gastric colonization, along with lower level infection of the intestine and colon, accompanied by persistent, low-level fecal shedding. The present study also demonstrated that melioidosis developed readily following low-dose oral inoculation with multiple different *B. pseudomallei* strains. In addition, we showed that *B. pseudomallei* colonized the GI tract readily following i.n. inoculation, but not following s.c. or i.p. inoculation.

The mouse model of enteric infection with *B. pseudomallei* exhibits a number of unique features when compared with enteric infection with other more well-known bacterial pathogens such as *Escherichia coli*, *Salmonella*, and *Shigella*. For example, the doses of *B. pseudomallei* required to infect mice orally (5×10^3^ to 5×10^4^ CFU) are relatively low compared with other enteric pathogens. The infectious doses reported for most *E. coli* and *Salmonella* strains in mice are in the range of 10^4^ to 10^9^ CFU [67], [68]. In addition, the level of enteric colonization and fecal shedding with *B. pseudomallei* was relatively low compared to other enteric pathogens. *Salmonella* and *E. coli* infection of mice results in fecal shedding titers ranging from 10^3^–10^8^ CFU/gram of feces [67], [68], [69], whereas fecal titers following *B. pseudomallei* infection of mice ranged from 10^2^–10^5^ CFU/gm feces, with an average titer of 10^3^ CFU/gm feces (see Figure 2).

Equally unique is the fact that *B. pseudomallei* appeared to preferentially colonize the stomach and not the lower GI tract. With *Salmonella* and enteropathogenic *E. coli* infection, the ileum and large intestine are the primary sites of bacterial replication and invasion [70], [71]. The only other well-known example of an enteric pathogen persistently colonizing the stomach is *H. pylori* [65]. However, unlike the case with *H. pylori* infection of the stomach, *B. pseudomallei* infection was not associated with any evidence of gastric inflammation, even after prolonged periods of infection. Thus, it is likely that *B. pseudomallei* has evolved resistance mechanisms to promote survival under harsh gastric conditions, and also to avoid immune clearance.

Another notable finding was that level of fecal shedding remained relatively stable for months in infected animals, with only minor fluctuations over time. In contrast, *E. coli*, *Salmonella* or *S. flexneri* fecal titers initially increase after inoculation and then decline to very low levels or disappear as the infection is controlled by the immune system [67], [68], [72]. Thus, in the *B. pseudomallei* model, the stable level of infection in the GI tract suggests a relative lack of local or systemic immune response against the organism. Moreover, the entire GI tract was infected in *B. pseudomallei* inoculated mice. This finding is quite remarkable considering the very different and inhospitable environments that exist within the stomach versus the small and large intestine.

It was also apparent that *B. pseudomallei* was an enteric colonizer rather than an invasive enteric pathogen. For example, infected mice did not develop detectable histological lesions at any site in the intestinal tract following sustained enteric infection. This was true even in mice subjected to very high challenge doses of *B. pseudomallei*, or in very susceptible strains of mice (eg, 129S6/SvEv mice). In contrast, infection with other enteric pathogens such as *Salmonella*, *S. dysenteriae*, and most *E. coli* strains produces significant intestinal pathology [73], [74]. Although enterotoxigenic *E. coli* infection can cause disease without inducing organ pathology, the toxins produced by this bacterium also result in diarrhea following infection [74]. However, mice with enteric *B. pseudomallei* infection did not exhibit signs of diarrhea or evidence of weight loss at any point during infection.

The lack of tissue invasion by *B. pseudomallei*, in either the stomach or intestine, likely explains both the lack of a gastric or intestinal inflammatory response or diarrhea in mice. The gastric mucosal lining is also where *H. pylori* colonizes the stomach, and microscopic niches within the gastric mucosa help *H. pylori* bacteria to avoid the low luminal pH [65]. While the pH in the lumen of the murine stomach is 3, the pH of the mucosa is thought to range from 4–6.5, and can be as high as 6.9 at epithelial surfaces [65], [75], [76]. *B. pseudomallei* is known to grow in broth culture at a pH of 4.5, and can increase the pH of broth media from 4.5 to 7 [77]. In humans the lumen of the human stomach typically has a pH of 2, but can vary from 2 to 5 [78], [79]. *B. pseudomallei* is known to survive in saline at a pH of 2 for one day, and has been isolated from surface water with a pH of 2 [20], [80]. Localization of *B. pseudomallei* to the stomach is also in agreement with previous case reports describing gastric ulcers in melioidosis patients [6], [37]. While reports of gastric ulcers are rare in melioidosis patients, the results of this study, as well as original experiments performed by Whitmore, and oral infection of horses with *B. mallei*, suggest that colonization without ulceration may be far more common than hitherto appreciated [25], [81]. Similar to *H. pylori* colonization, gastric disease may occur only if *B. pseudomallei* becomes invasive [65].

Major risk factors associated with development of melioidosis in humans include diabetes, alcoholism and chronic kidney disease [66]. Interestingly, patients with diabetes, alcoholism and chronic kidney disease also frequently develop gastrointestinal lesions, particularly gastric lesions. For example, dyspepsia is a common symptom in diabetics, and diabetic patients are known to develop gastric ulcers more frequently than non-diabetics [82], [83]. In addition, diabetics can develop gastric ulcers, erosions and severe acute gastritis without dyspepsia symptoms [84]. Excessive alcohol consumption is known to directly damage the mucosa of the esophagus, stomach and small intestine, with exposure to high concentrations resulting in gastric hemorrhaging [85], [86], [87]. Excessive alcohol consumption can increase intestinal permeability and toxin release, leading to increased risk for infection [87], [88]. Finally, the association between GI pathology and chronic kidney disease is well described, and patients with chronic kidney disease have a higher frequency of upper GI tract lesions than the general population [89], [90], [91], [92]. Additionally, GI bleeding occurs in 19% of chronic kidney disease patients, and 61% of these lesions were localized to the duodenum [93]. Thus, multiple factors associated with increased risk of disseminated melioidosis are also associated with increased risk of gastric lesions, which could provide an avenue for increased dissemination of *B. pseudomallei* from the colonized gastric mucosa.

Since enteric colonization with *B. pseudomallei* did not produce signs of GI disease in infected mice (and may not in humans as well), the GI tract could well be considered a sanctuary for persistent subclinical infection with *B. pseudomallei*. If the organism can maintain chronic low level, subclinical enteric infection, it may persist undetected for months or years. This could then account for the long lag between initial exposure to the organism and the development of overt disseminated disease to extra-intestinal sites. Alternatively, over time low numbers of the organism may spontaneously enter the bloodstream, leading eventually to organ seeding and disseminated infection. In this model, the relative risk of developing disseminated *B. pseudomallei* infection would increase with the absolute duration of enteric infection. Although a carrier state of *B. pseudomallei* in humans has not been identified thus far, only throat swabs have been evaluated as a screening test [94], [95].

Oral colonization may be overlooked due to multiple technical challenges involved with processing GI samples, as well as the low level of *B. pseudomallei* shedding that may occur. For instance, previous studies have shown that enteric bacteria from fecal swabs often outcompete *B. pseudomallei* when grown on Ashdown's medium [31]. In our current study, in order to increase the sensitivity of organism detection, we had to develop a more selective *Burkholderia* culture medium, and we also processed large volumes of fecal material per mouse at each sampling point. A similar approach may be difficult in humans, and may require culture in enrichment broth or concentration prior to analysis. Alternatively, molecular or fluorescent techniques may be necessary to identify *B. pseudomallei* in fecal samples. For example, molecular techniques were used to demonstrate *B. pseudomallei* in the feces of grazing animals [96].

Further studies will be needed to determine how *B. pseudomallei* disseminates from the GI tract. In the current study *B. pseudomallei* was only rarely isolated from mesenteric lymph nodes, unlike the case with *Salmonella* and *Y. enterocolitica* following GI tract infection [53], [55], [56], [57]. Furthermore, unlike infection with *Salmonella* or *Shigella*, the gall bladder was rarely colonized with *B. pseudomallei* [54], [55], [58]. Because FISH experiments localized *B. pseudomallei* to the stomach, dissemination may be occurring through the gastric lymph node. This would be in agreement with isolation of *B. pseudomallei* from the gastric lymph node of monkeys, and the gastrohepatic lymph node of pigs [29], [97]. Another possibility is that *B. pseudomallei* may be disseminating from the ileum, as both *B. pseudomallei* and neutrophil recruitment were localized to the ileum by FISH and histopathology experiments. Although both of these observations were rare, the identification of both exclusively in the ileum warrants further investigation.

Studies were also conducted to accurately localize bacteria immediately after oral inoculation (see Methods). Immediately following oral inoculation with 5×10^5^ CFU *B. pseudomallei*, 21% of mice had *B. pseudomallei* in the lungs (limit of detection = 4 CFU/lung). This rate of inadvertent aspiration is much lower than the 89% of mice which ultimately went on to develop disseminated infection following oral infection with Bp1026b (5×10^5^ CFU) (Figure 4). Therefore, the observations in our study are unlikely to be due solely to aspiration during oral inoculation.

Many of the findings from our mouse model of chronic *B. pseudomallei* enteric infection need to be evaluated in humans with chronic *B. pseudomallei* infection to determine whether similar rates and locations of enteric infection also occur in humans. For example, patients with melioidosis as well as asymptomatic patients from melioidosis endemic regions could be screened using sensitive assays combined with endoscopic gastric biopsies and repeated cultures for detection of low-level fecal shedding of the organism. If persistent enteric colonization with *B. pseudomallei* was found to be prevalent in humans, such a finding would have significant implications for understanding human melioidosis and developing public health efforts to control or prevent infection.

In summary, our findings in a mouse model of oral inoculation with *B. pseudomallei* indicate clearly that this organism is an efficient colonizer of the GI tract. Therefore, genes associated with environmental survival by *B. pseudomallei* may also be important for survival in the GI tract [98]. Understanding the mechanisms that *B. pseudomallei* uses to sustain persistent enteric colonization may also yield important insights into how the organism disseminates from the GI tract to organs such as the spleen, liver, and central nervous system.

## Materials and Methods

### Ethics statement

This study was carried out in strict accordance with the recommendations in the Guide for the Care and Use of Laboratory Animals of the National Institutes of Health. The animal use protocol was approved by the Institutional Animal Care and Use Committee at Colorado State University (Animal Welfare Assurance Number A3572-01). Infected animals were monitored closely three times daily and all efforts were made to minimize suffering.

### Mice

BALB/c and C57BL/6 mice were purchased from Jackson Laboratories (Bar Harbor, ME). 129S6/SvEv mice were purchased from Taconic Laboratories (Germantown, NY), and ICR mice were purchased from Harlan Laboratories (Indianapolis, IN). All mice used in experiments were housed under pathogen-free conditions in micro-isolator cages and mice were 6–10 weeks of age at the time of infection. All experiments involving animals were approved by the Institutional Animal Care and Use Committee at Colorado State University.

### Bacteria

*B. pseudomallei* strain 1026b (Bp1026b) is a clinical isolate recovered from the blood of a human patient with septicemic melioidosis in Thailand [99]. Three low-passage clinical *B. pseudomallei* isolates recovered from melioidosis patients in Thailand were also used in this study. Strain 2671a (Bp2671a) was isolated from blood culture, while strain 2685a (Bp2685a) was isolated from a pus sample, and strain 2719a (Bp2719a) was isolated from the lungs. In BSL-2 experiments the purM^−^ mutant *B. pseudomallei* strain 82 (Bp82) derived from Bp1026b was used [50]. For *B. thailandensis* experiments, strain E264, an environmental isolate from Thailand was used [100]. All strains were grown in LB broth (BD Biosciences, San Jose, CA), and stationary phase cultures were frozen at −80°C in LB broth +20% glycerol (Fisher Scientific, Pittsburgh, PA). All experiments were performed with strains from a single freezing event. All procedures involving *B. pseudomallei* were performed in a Biosafety Level 3 (BSL3) facility, in accordance with approved BSL3 and Select Agent protocols in place at Colorado State University.

### Animal infections

Immediately prior to animal inoculation bacterial stocks that had been frozen in LB broth with 20% glycerol were thawed and diluted in sterile phosphate buffered saline (PBS) (Sigma-Aldrich, St. Louis, MO). Infectious doses were determined by plating serial dilutions of each inoculum on LB agar (BD Biosciences). Oral (p.o.) inoculations were done using a stainless steel 22 gauge gavage needle and mice were inoculated using a total volume of 100 µl. For intranasal (i.n.) inoculation, mice were anesthetized with intraperitoneal (i.p.) injection of ketamine (100 mg/kg) (Pfizer, New York, NY), and xylazine (10 mg/kg) (Lloyd Laboratories, Shenandoah, IA). Intranasal inoculations were done using a volume of 20 µl (10 µl per nostril). Subcutaneous (s.c) inoculations were done in the right groin and mice were inoculated with a total volume of 100 µl. Intraperitoneal inoculation was done by i.p. injection in a total volume of 200 µl.

LD_50_ values for acute disease (ie, euthanasia required on or before day 7) were calculated using the Reed-Muench method [101]. LD_50_ values in BALB/c used in this study include 3.8×10^7^ CFU for s.c. inoculation, 1.7×10^6^ CFU for i.p. inoculation, and as described previously, 9×10^2^ CFU for i.n. inoculation [102]. Preliminary experiments were performed to determine if pulmonary infection occurs following oral infection due to inadvertent aspiration of bacteria during oral gavage with *B. pseudomallei*. Following p.o. inoculation with 5×10^5^ CFU *B. pseudomallei*, BALB/c mice were euthanized two hours after infection and bacterial burdens were determined in the lungs as described below (Limit of detection = 4 CFU/organ). On average *B. pseudomallei* was cultured from the lungs of 3/14 (21%) mice (data pooled from 3 independent experiments). These results are similar to previous studies, and due to the potential for aspiration into the lungs following p.o. inoculation, any mouse succumbing to acute disease with higher bacterial burdens in the lung than in GI tissues was excluded from the analysis [44].

Previous studies in our laboratory have demonstrated that following i.n. inoculation, 40% of the inoculum reaches the lungs [103]. Briefly, BALB/c mice (n = 9) were inoculated i.n. as described above, and pulmonary bacterial burdens were determined 3 hours after infection.

### Selective medium for isolation of *B. pseudomallei* from gastrointestinal tissues

The selective medium used most often for isolation of *B. pseudomallei* from clinical samples is Ashdown's medium (ASH) [49], [104], [105]. In preliminary studies we found that ASH failed to prevent the growth of normal gut commensal bacteria and that these bacteria in many cases outcompeted *B. pseudomallei*. Therefore, to suppress the growth of commensal bacteria we added norfloxacin, ampicillin, and polymyxin B to ASH media (NAP-A) for selective isolation of *B. pseudomallei* from intestinal contents and feces, based on previously reported media and antibiotic susceptibility profiles of *B. pseudomallei* [104], [105], [106], [107]. To prepare NAP-A medium, we used ASH medium as the basal medium [49]. Briefly, 4% glycerol, 5 µg/ml crystal violet (EMD Science, Gibbstown, NJ), 50 µg/ml neutral red (Sigma-Aldrich), and 4 µg/ml gentamicin (Sigma-Aldrich) were added to trypticase soy agar (BD Biosciences). After ASH was autoclaved and cooled to 50–60°, norfloxacin (4 µg/ml) (Sigma-Aldrich), ampicillin (10 µg/ml) (Sigma-Aldrich) and polymyxin B (300 units/ml) (Sigma-Aldrich) were added to prepare NAP-A medium.

### Determination of sepsis and organ bacterial burden

For quantitative blood culture, serial 10-fold dilutions of heparinized blood were diluted in sterile PBS and dilutions were plated on LB agar plates. Bacterial burden in organ homogenates was quantitated as described previously, with slight modifications for the isolation of *B. pseudomallei* from GI organs [102]. Mice were euthanized and organs were placed in 4 ml sterile PBS. To ensure efficient homogenization, the stomach and cecum were cut into ∼1–2 cm^2^ sections, while small intestine and colon tissues were cut open longitudinally, and then cut into 2–3 cm lengths. Organs were homogenized using a Stomacher 80 Biomaster (Seward, Bohemia, NY) and serial 10-fold dilutions of organ homogenates were prepared in sterile PBS. Lung, liver, spleen, kidney, brain, gall bladder, and mesenteric lymph node homogenates were plated on LB agar plates. Stomach, SI, cecum, and colon homogenates were plated on NAP-A agar (described above). All agar plates were incubated at 37°C and colonies were counted at 48 hours. The limit of detection in blood was 10 CFU/ml, while the limit of detection in organ homogenates ranged from 1–20 CFU/organ.

### Isolation of *B. pseudomallei* from fecal pellets

Fecal pellets were collected by transferring mice from their cage into a plastic container, where the pellets were collected and placed in sterile PBS at a concentration of 0.1 gram feces per ml PBS. Multiple fecal pellets from each mouse (typically 5–6 pellets per mouse) were homogenized using a Stomacher 80 Biomaster. Serial dilutions of fecal homogenates were prepared in sterile PBS and plated on NAP-A agar plates. The limit of bacterial detection in feces was 10–60 CFU/gram of feces.

### Fluorescent *in situ* hybridization

Fluorescent *in situ* hybridization (FISH) was performed using antisense ssDNA probes targeting the bacterial 16S rRNA. *B. pseudomallei* specific probes used in this study were designed in our laboratory. Two probes designated Bpm427 (5′-CCACTCCGGGTATTAGCCAGA-3′) (positions 427 to 447) and Bpm975 (5′-CGCCCAACTCTCATCGGG-3′) (positions 975 to 992) were identified based on binding regions on the 16S rRNA gene of *B. pseudomallei* strain 1026b. Probe specificity was confirmed in preliminary experiments performed on bacterial cultures which demonstrated that both Bpm427 and Bpm975 bound to Bp82 [50], and *B. mallei* ATCC23344 but not to *B. thailandensis* E264 or fecal bacteria (data not shown). Consistent with previous reports, we were unable to develop probes capable of differentiating *B. mallei* and *B. pseudomallei* [108], [109], [110]. The previously described Eub338 probe (5′-GCTGCCTCCCGTAGGAGT-3′) which recognizes a conserved sequence present in the 16S rRNA of all bacteria, and the irrelevant Non338 probe (5′-ACTCCTACGGGAGGCAGC-3′) containing a sequence complementary to the Eub338 probe were also used [111], [112]. All probes were purchased from Integrated DNA Technologies (San Diego, CA). Bpm427 and Bpm975 probes were 5′ labeled with Cy3, and Eub338 and Non338 probes were 5′ labeled with 6-FAM.

Tissue fixation was performed as described previously [102]. Briefly, tissues were placed in 10% neutral buffered formalin (NBF) (Sigma-Aldrich) for 48 hours. The entire small intestine was collected as a “Swiss roll” and fixed in 10% NBF for 48 hours. After 48 hours in 10% NBF, all organs were transferred into a solution of 70% ethanol for 7 days. Tissues were then embedded in paraffin, and sectioned.

FISH was performed as described previously [113]. Prior to performing the FISH assay tissue sections were baked for 1 hour at 60°C. Sections were then deparaffinized with Histoclear® (National Diagnostics, Atlanta, GA) and re-hydrated in solutions with decreasing ethanol concentration. Sections were post-fixed in 4% paraformaldehyde (Electron Microscopy Science, Hatfield, PA) in PBS for 15 minutes at room temperature and washed in PBS. Tissue sections were then permeabilized using one of two proteinase K digestion (PK) protocols. PCR grade PK was purchased from Roche (Indianapolis, IN) and was diluted in 10 mM Tris pH 7.5, 5 mM CaCl_2_, and 0.2% Triton X-100 (All reagents from Fisher Scientific, Pittsburgh, PA). Preliminary experiments were performed to determine the optimal digestion conditions resulting in maximal signal strength from enteric bacteria, or the maximal digestion procedure which did not alter tissue morphology. Maximal signal from enteric bacteria was obtained following digestion in 20 µg/ml PK for 30 minutes at 37°C, as accessed by signal intensity following hybridization with the Eub338 probe. In contrast, the maximal PK digestion which did not alter tissue morphology was determined to be 5 µg/ml PK for 8 minutes at 37°C, as accessed by changes in nuclear morphology following DAPI staining (data not shown). Therefore, for identification of *B. pseudomallei* in ingesta of the stomach and cecum and fecal material in the colon, sections were digested in 20 µg/ml PK for 30 minutes at 37°C. For localization of *B. pseudomallei* in tissues, a separate set of sections was digested in 5 µg/ml PK for 8 minutes at 37°C. All small intestine sections were digested in 5 µg/ml PK for 8 minutes at 37°C, as preliminary experiments demonstrated that no increase in signal from enteric bacteria in the ingesta of the small intestine was observed regardless of the PK digestion protocol used (data not shown). Following PK digestion, sections were washed in 30 mM glycine (Fisher Scientific) to stop proteolysis, followed by a PBS wash. Next, tissue sections were hybridized with ssDNA probes. Probes were diluted in hybridization buffer consisting of 4× saline sodium citrate (SSC) (Fisher Scientific), 200 mg/ml dextran sulfate (Sigma, St. Louis, MO), 20% formamide (Sigma), 0.25 mg/ml PolyA (Sigma), 0.25 mg/ml salmon sperm DNA (Invitrogen, Carlsbad, CA), 0.25 mg/ml tRNA (Invitrogen), and 0.5× Dendhart's solution (Sigma). Slides were hybridized with a cocktail of the Bpm427, Bpm975 and Eub338 probes each used at a final concentration of 1 µg/ml, or the Non-338 probe at a final concentration of 3 µg/ml. Probes were hybridized with tissue sections in a humidified chamber at 37°C for 24 hours. Following hybridization sections were washed to remove non-specific probe binding. Washes included, one 15 minute wash in 1× SSC at 37°C, two 15 minute washes in 1× SSC at 55°C, two 15 minute washes in 0.5× SSC at 55°C, and one 10 minute wash in 0.5× SSC at room temperature. Slides were washed in dH_2_O at room temperature for two minutes, air dried, and mounted with Pro-Long gold containing DAPI (Invitrogen).

### Fluorescent microscopy

Following hybridization with FISH probes tissue sections were observed at 1000× final magnification using an Olympus BX51 fluorescent microscope (Olympus, Center Valley, PA) with DAPI (Ex. 377/50 nm; Dichroic 409 nm; Em. 477/60 nm), FITC (Ex. 482/35 nm; Dichroic 506 nm; Em. 536/40 nm) and Cy3 (Ex. 531/40 nm; Dichroic 562 nm; Em. 593/40 nm) filter sets (Semrock, Lake Forest, IL). Photomicrographs were captured with a DP71 camera using CellSens Entry software version 1.5 (Olympus, Center Valley, PA). Fluorescent overlays were created by combining individual fluorescent images in Photoshop CS3 software (Adobe, San Jose, CA). When necessary multiple images were obtained at different focal planes and combined using layer masks in Photoshop software. All other manipulations were applied to the images globally. Settings used on images obtained from tissues hybridized with the Bpm427, Bpm975 and Eub338 probes were determined from images captured from tissues hybridized with the Non338 probe.

### Histological analysis

Tissue processing, staining, and analysis of organ pathology were performed as described previously [102]. Tissues were processed as described above for FISH analysis, and lungs were inflated with 10% NBF via the trachea for 5 minutes prior to removal, and then placed in 10% NBF for 48 hours. After fixation tissues were embedded in paraffin, sectioned, and stained with hematoxylin and eosin. Tissues were examined by a veterinary pathologist (H.B.O.) experienced in mouse pathology. Photomicrographs were taken using a Nikon Eclipse 51E microscope and a Nikon DS-Fi1 camera with a DS-U2 unit and NIS elements F software and optimized using Photoshop CS3 software (Adobe) with all changes applied globally.

### Statistical analysis

Statistical analyses were done using Prism 5.0 software (Graph Pad, San Diego, CA). Analyses comparing two groups were done using a two-tailed Student's t-test, and analyses comparing more than two groups were performed using a one-way ANOVA followed by a Tukey's multiple means comparison test. Differences in percentages of positive samples were compared using a two-tailed Fisher's exact test. Differences were considered statistically significant for *p*<0.05, and statistical trends were considered for *p*<0.1.

## Supporting Information

Figure S1 **Bacterial dissemination to systemic organs following oral inoculation with 3** ***B. pseudomallei*** **clinical isolates.** BALB/c mice (n = 9–11 animals evaluated per bacterial strain) were inoculated orally with Bp2671a (3.6×10^5^ CFU); Bp2685a (2.9×10^5^ CFU); or Bp2719a (3.5×10^5^ CFU). At day 3 after infection, organs were processed for determination of bacterial burden as described in Materials and Methods. Data are presented as individual values with solid bars representing the mean log_10_ titer. Organ bacterial burdens are expressed as log_10_ CFU/organ, and blood titers are graphed as log_10_ CFU/ml. Dashed bars represent the mean log_10_ titers from day 3 Bp1026b bacterial burden determination (Reproduced from Figure 4 for reference). Data were pooled from 2 independent experiments. The limit of detection was 20 CFU/organ, and 10 CFU/ml for blood. Statistical differences were determined between Bp1026b and each clinical strain using a two tailed Student's t-test.
(TIF)

Figure S2 **Localization of** ***B. pseudomallei*** **2671a in gastrointestinal organs following oral infection.** Stomach (A), small intestine (B), cecum (C) and colon (D) tissues from mice infected orally with 2.0×10^4^ CFU *B. pseudomallei* strain 2671a were collected 21 days after infection. FISH was performed on tissue sections as described in Materials and Methods. Tissue sections were counterstained with DAPI (blue) and observed at 1000× final magnification. Tissue sections were hybridized with a eubacterial probe (green), and two *B. pseudomallei* specific probes (red). Arrows in B–D indicate the location of *B. pseudomallei*. In all images the scale bar represents 10 microns.
(TIF)

Figure S3 **Localization of** ***B. pseudomallei*** **2685a in gastrointestinal organs following oral infection.** Stomach (A), small intestine (B), cecum (C) and colon (D) tissues from mice infected orally with 4.8×10^4^ CFU *B. pseudomallei* strain 2685a were collected 3 days after infection. FISH was performed on tissue sections as described in Materials and Methods, and sections were counterstained with DAPI (blue) and observed at 1000× final magnification. Tissue sections were hybridized with a eubacterial probe (green), and two *B. pseudomallei* specific probes (red). Arrows in C and D indicate the location of *B. pseudomallei*. In all images the scale bar represents 10 microns.
(TIF)

Figure S4 **Localization of** ***B. pseudomallei*** **2719a in gastrointestinal organs following oral infection.** Stomach (A), small intestine (B), cecum (C) and colon (D) tissues from mice infected orally with 2.8×10^4^ CFU *B. pseudomallei* strain 2719a were collected 4 days after infection. FISH was performed on tissue sections as described in Materials and Methods, sections were counterstained with DAPI (blue) and observed at 1000× final magnification. Tissue sections were hybridized with a eubacterial probe (green), and two *B. pseudomallei* specific probes (red). Arrows in B–D indicate the location of *B. pseudomallei*. In all images the scale bar represents 10 microns.
(TIF)

Figure S5 **Localization of** ***B. pseudomallei*** **colonization in the stomach.** BALB/c mice were infected orally with *B. pseudomallei* strain 1026b (5×10^5^ CFU), Bp2671a (2.0×10^4^ CFU), Bp2685a (4.8×10^4^ CFU) or Bp2719a (2.8×10^4^ CFU). Stomach tissues were collected from Bp1026b mice 56 days after infection, Bp2671a mice 21 days after infection, Bp2685a mice 3 days after infection, and Bp2719a mice 4 days after infection. FISH was performed on stomach tissue sections as described in Materials and Methods. Tissues were counterstained with DAPI and observed at 1000× final magnification. Positive 1000× fields containing *B. pseudomallei* from mice infected with Bp1026b (A), Bp2671a (B), Bp2685a (C) or Bp719a (D) are indicated by white outlines. Outlines are overlaid onto stomach images created by combining images of DAPI staining obtained from each stomach. The esophagus (esoph.), body (corpus) and pylorus of the stomach are labeled for reference. The scale bar in all images represents 2 mm.
(TIF)

Figure S6 **Mice lack gastric pathology following oral infection with different** ***B. pseudomallei*** **isolates.** BALB/c mice were infected orally with *B. pseudomallei* strain Bp1026b (5×10^5^ CFU), Bp2671a (2.0×10^4^ CFU), Bp2685a (4.8×10^4^ CFU) or Bp2719a (2.8×10^4^ CFU). Stomach tissues were collected from Bp1026b mice 56 days after infection, Bp2671a mice 21 days after infection, Bp2685a mice 3 days after infection, and Bp2719a mice 4 days after infection. All tissues were fixed in 10% NBF, embedded in paraffin and stained with hematoxylin and eosin. Representative stomach images from Bp1026b (A), Bp2671a (B), Bp2685a (C), and Bp2719a (D) are shown. The location of each image within the stomach is indicated by a star on the representative stomach image in the bottom left corner of each image. Images were captured at 400× final magnification, and the scale bar on all images represents 25 microns.
(TIF)
