# Supplementary figures and images for: Persistent Gastric Colonization with Burkholderia pseudomallei and Dissemination from the Gastrointestinal Tract following Mucosal Inoculation of Mice
Source: PLoS One. 2012 May 18;7(5):e37324. doi: 10.1371/journal.pone.0037324 (PMC3356274; doi:10.1371/journal.pone.0037324)

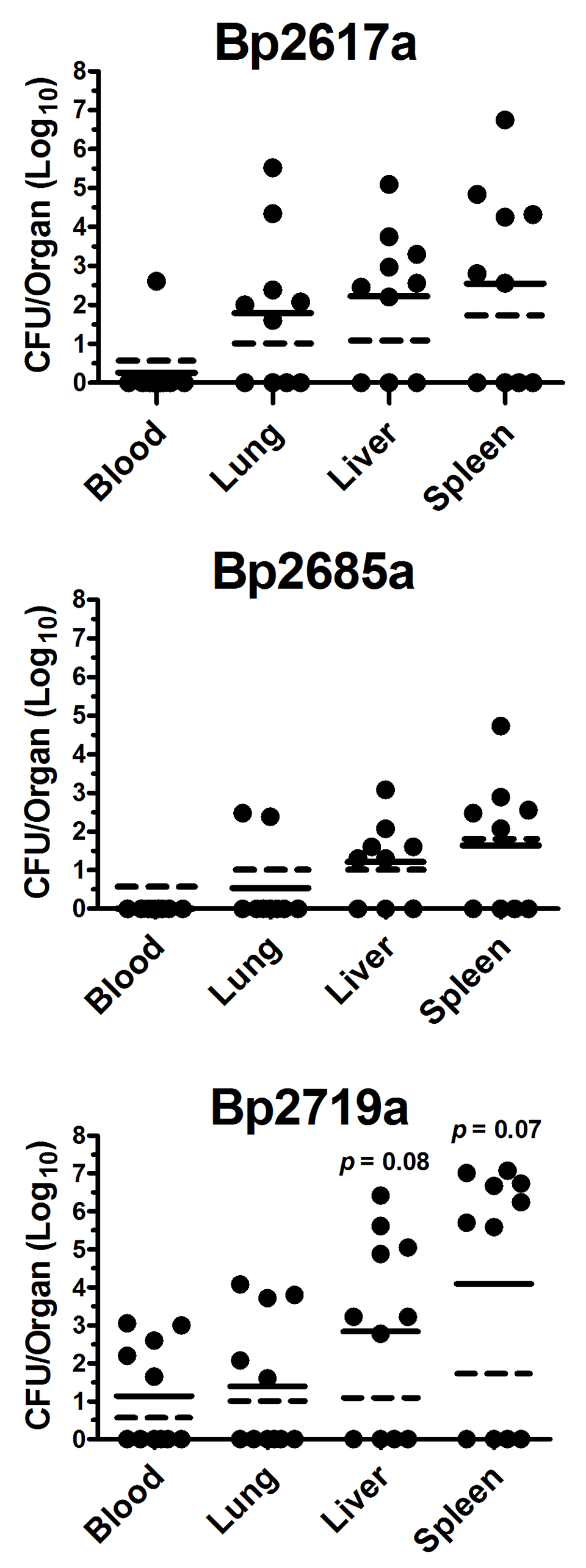

Supplement: Figure S1 — Bacterial dissemination to systemic organs following oral inoculation with 3 B. pseudomallei clinical isolates. BALB/c mice (n = 9–11 animals evaluated per bacterial strain) were inoculated orally with Bp2671a (3.6×10^5^ CFU); Bp2685a (2.9×10^5^ CFU); or Bp2719a (3.5×10^5^ CFU). At day 3 after infection, organs were processed for determination of bacterial burden as described in Materials and Methods. Data are presented as individual values with solid bars representing the mean log10 titer. Organ bacterial burdens are expressed as log10 CFU/organ, and blood titers are graphed as log10 CFU/ml. Dashed bars represent the mean log10 titers from day 3 Bp1026b bacterial burden determination (Reproduced from Figure 4 for reference). Data were pooled from 2 independent experiments. The limit of detection was 20 CFU/organ, and 10 CFU/ml for blood. Statistical differences were determined between Bp1026b and each clinical strain using a two tailed Student's t-test. (TIF) [file pone.0037324.s001.tif]

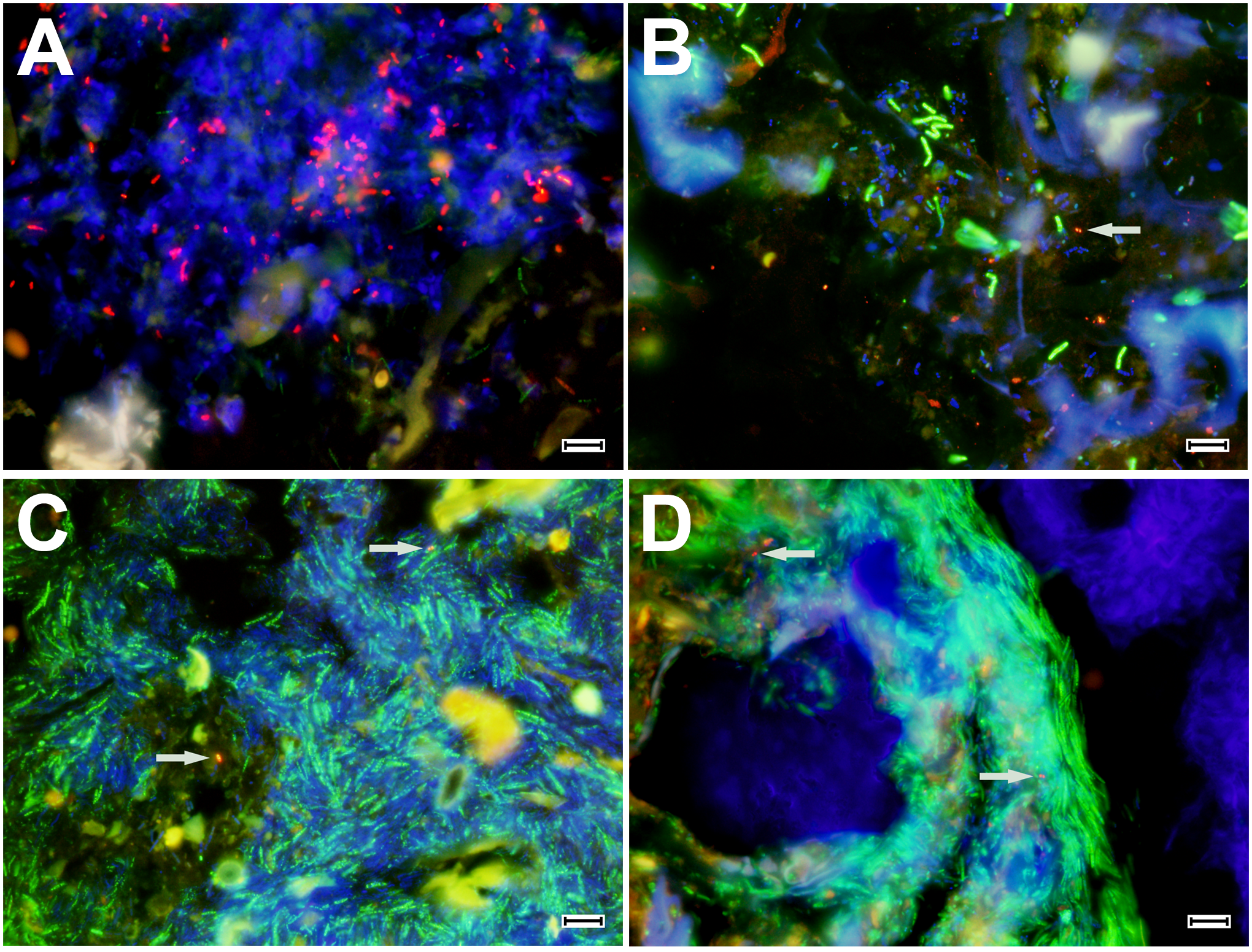

Supplement: Figure S2 — Localization of B. pseudomallei 2671a in gastrointestinal organs following oral infection. Stomach (A), small intestine (B), cecum (C) and colon (D) tissues from mice infected orally with 2.0×10^4^ CFU B. pseudomallei strain 2671a were collected 21 days after infection. FISH was performed on tissue sections as described in Materials and Methods. Tissue sections were counterstained with DAPI (blue) and observed at 1000× final magnification. Tissue sections were hybridized with a eubacterial probe (green), and two B. pseudomallei specific probes (red). Arrows in B–D indicate the location of B. pseudomallei. In all images the scale bar represents 10 microns. (TIF) [file pone.0037324.s002.tif]

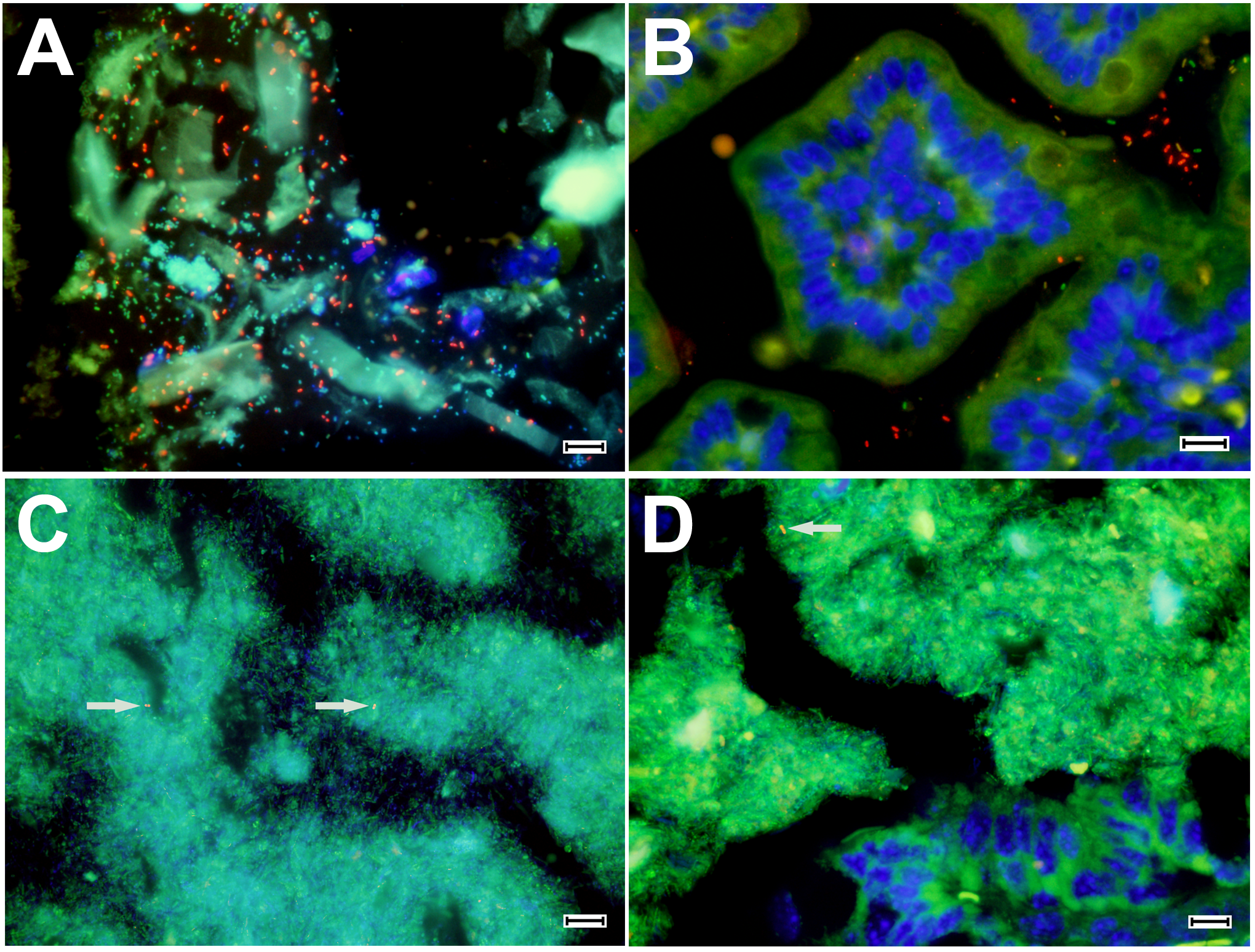

Supplement: Figure S3 — Localization of B. pseudomallei 2685a in gastrointestinal organs following oral infection. Stomach (A), small intestine (B), cecum (C) and colon (D) tissues from mice infected orally with 4.8×10^4^ CFU B. pseudomallei strain 2685a were collected 3 days after infection. FISH was performed on tissue sections as described in Materials and Methods, and sections were counterstained with DAPI (blue) and observed at 1000× final magnification. Tissue sections were hybridized with a eubacterial probe (green), and two B. pseudomallei specific probes (red). Arrows in C and D indicate the location of B. pseudomallei. In all images the scale bar represents 10 microns. (TIF) [file pone.0037324.s003.tif]

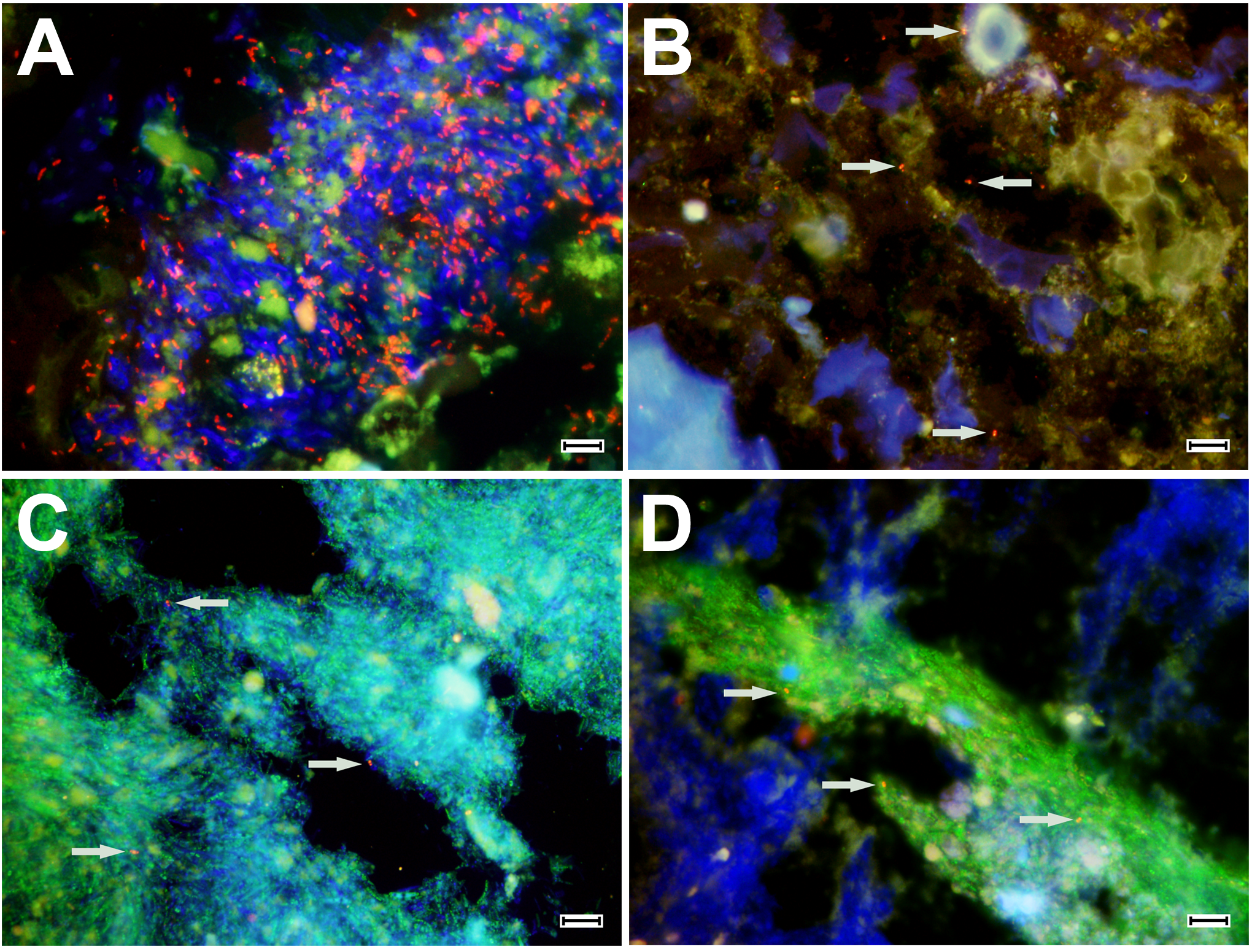

Supplement: Figure S4 — Localization of B. pseudomallei 2719a in gastrointestinal organs following oral infection. Stomach (A), small intestine (B), cecum (C) and colon (D) tissues from mice infected orally with 2.8×10^4^ CFU B. pseudomallei strain 2719a were collected 4 days after infection. FISH was performed on tissue sections as described in Materials and Methods, sections were counterstained with DAPI (blue) and observed at 1000× final magnification. Tissue sections were hybridized with a eubacterial probe (green), and two B. pseudomallei specific probes (red). Arrows in B–D indicate the location of B. pseudomallei. In all images the scale bar represents 10 microns. (TIF) [file pone.0037324.s004.tif]

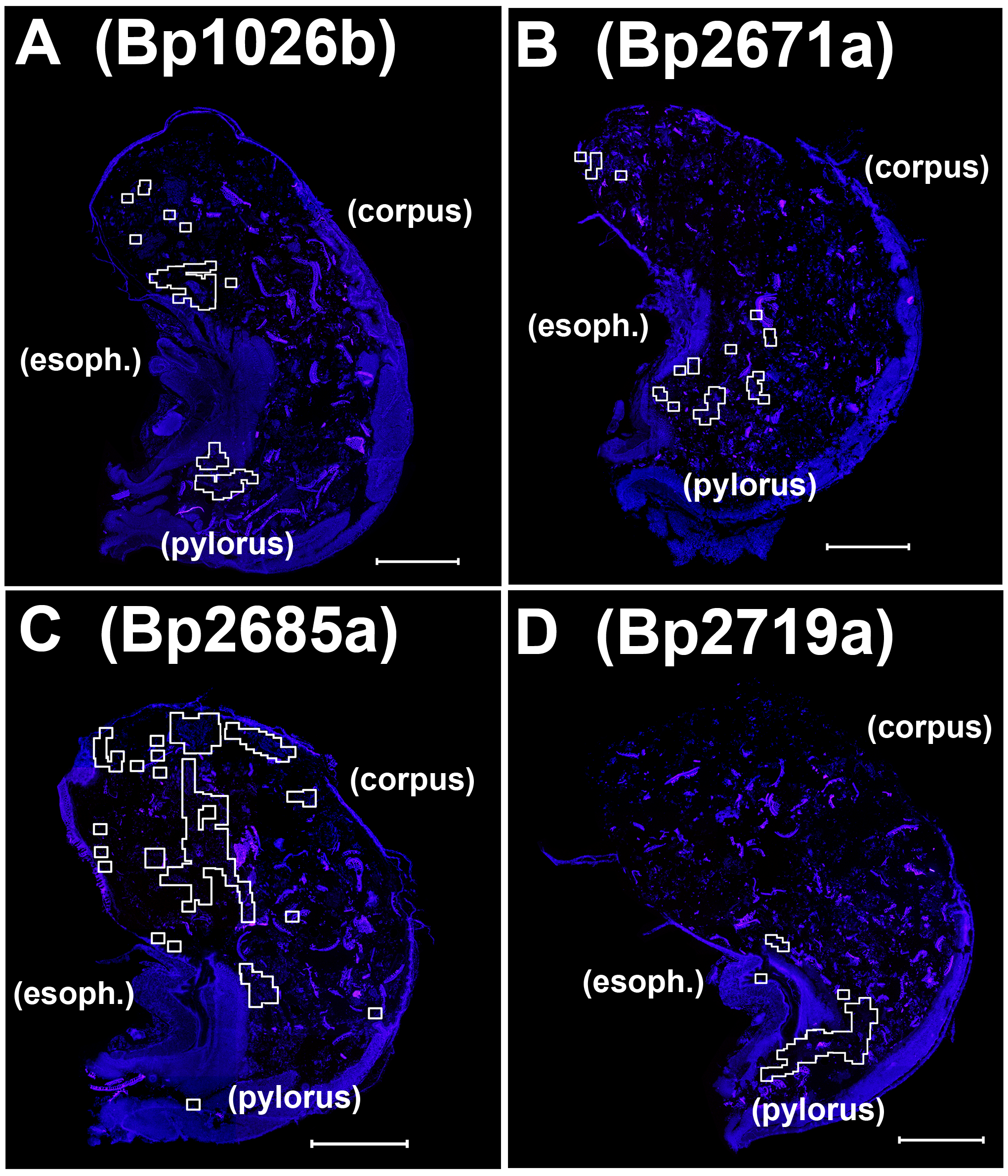

Supplement: Figure S5 — Localization of B. pseudomallei colonization in the stomach. BALB/c mice were infected orally with B. pseudomallei strain 1026b (5×10^5^ CFU), Bp2671a (2.0×10^4^ CFU), Bp2685a (4.8×10^4^ CFU) or Bp2719a (2.8×10^4^ CFU). Stomach tissues were collected from Bp1026b mice 56 days after infection, Bp2671a mice 21 days after infection, Bp2685a mice 3 days after infection, and Bp2719a mice 4 days after infection. FISH was performed on stomach tissue sections as described in Materials and Methods. Tissues were counterstained with DAPI and observed at 1000× final magnification. Positive 1000× fields containing B. pseudomallei from mice infected with Bp1026b (A), Bp2671a (B), Bp2685a (C) or Bp719a (D) are indicated by white outlines. Outlines are overlaid onto stomach images created by combining images of DAPI staining obtained from each stomach. The esophagus (esoph.), body (corpus) and pylorus of the stomach are labeled for reference. The scale bar in all images represents 2 mm. (TIF) [file pone.0037324.s005.tif]

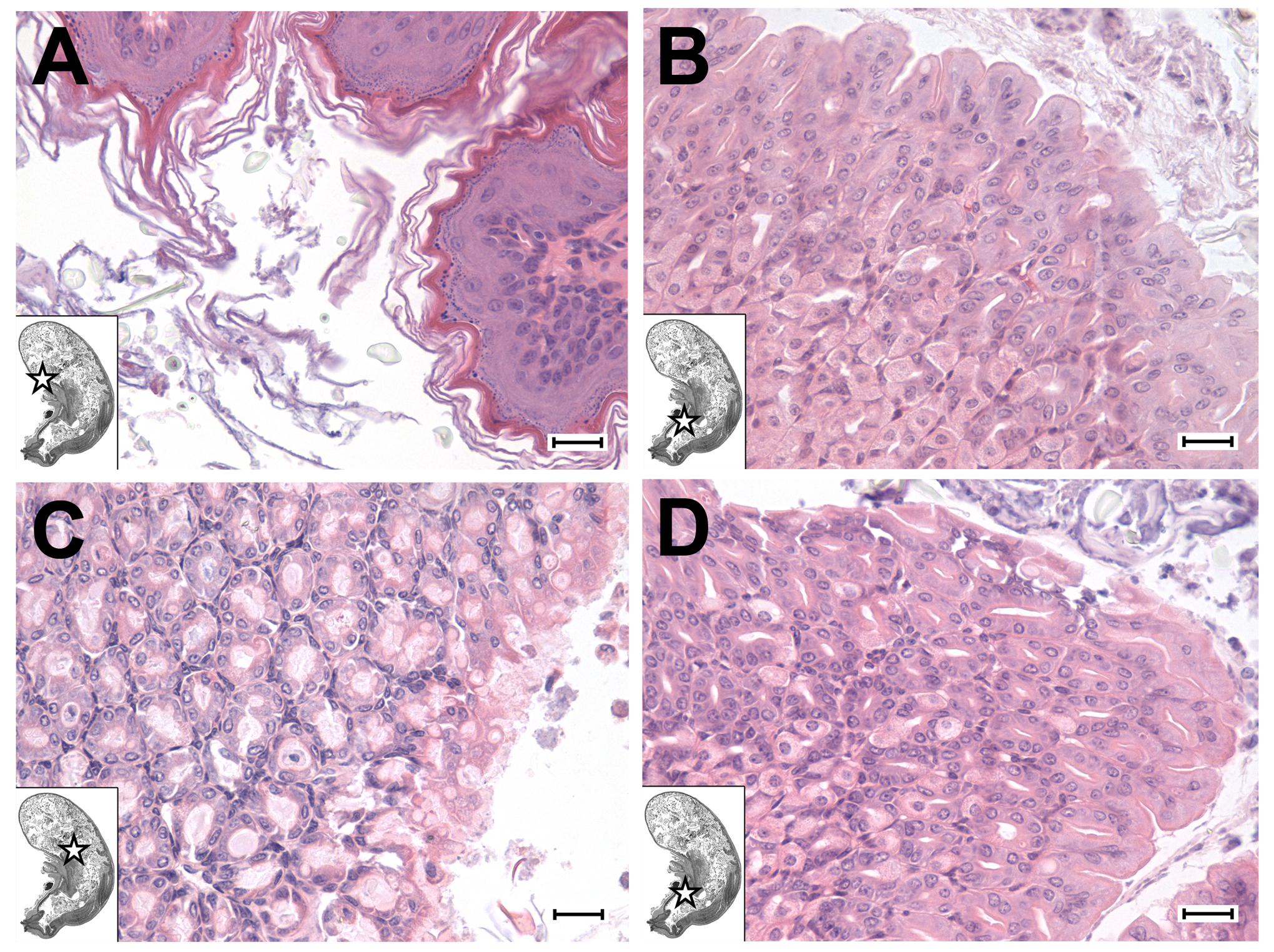

Supplement: Figure S6 — Mice lack gastric pathology following oral infection with different B. pseudomallei isolates. BALB/c mice were infected orally with B. pseudomallei strain Bp1026b (5×10^5^ CFU), Bp2671a (2.0×10^4^ CFU), Bp2685a (4.8×10^4^ CFU) or Bp2719a (2.8×10^4^ CFU). Stomach tissues were collected from Bp1026b mice 56 days after infection, Bp2671a mice 21 days after infection, Bp2685a mice 3 days after infection, and Bp2719a mice 4 days after infection. All tissues were fixed in 10% NBF, embedded in paraffin and stained with hematoxylin and eosin. Representative stomach images from Bp1026b (A), Bp2671a (B), Bp2685a (C), and Bp2719a (D) are shown. The location of each image within the stomach is indicated by a star on the representative stomach image in the bottom left corner of each image. Images were captured at 400× final magnification, and the scale bar on all images represents 25 microns. (TIF) [file pone.0037324.s006.tif]
